# Post-weaning epiphysiolysis causes distal femur dysplasia and foreshortened hindlimbs in fetuin-A-deficient mice

**DOI:** 10.1371/journal.pone.0187030

**Published:** 2017-10-31

**Authors:** Laura J. Brylka, Sina Köppert, Anne Babler, Beate Kratz, Bernd Denecke, Timur A. Yorgan, Julia Etich, Ivan G. Costa, Bent Brachvogel, Peter Boor, Thorsten Schinke, Willi Jahnen-Dechent

**Affiliations:** 1 Biointerface Laboratory, Helmholtz Institute for Biomedical Engineering, RWTH Aachen University Hospital, Aachen, Germany; 2 IZKF Genomics Facility, Interdisciplinary Center for Clinical Research, RWTH Aachen University Hospital, Aachen, Germany; 3 Department of Osteology and Biomechanics, University Medical Center Hamburg-Eppendorf, Hamburg, Germany; 4 Department of Pediatrics and Adolescent Medicine, Experimental Neonatology, Medical Faculty, University of Cologne, Cologne, Germany; 5 Center for Biochemistry, Medical Faculty, University of Cologne, Cologne, Germany; 6 IZKF Research Group Computational Biology and Bioinformatics, Interdisciplinary Center for Clinical Research, RWTH Aachen University Hospital, Aachen, Germany; 7 Department of Pathology & Division of Nephrology and Immunology, RWTH Aachen University Hospital, Aachen, Germany; Nanjing Medical University, CHINA

## Abstract

Fetuin-A / α_2_-Heremans-Schmid-glycoprotein (gene name *Ahsg*) is a systemic inhibitor of ectopic calcification. Due to its high affinity for calcium phosphate, fetuin-A is highly abundant in mineralized bone matrix. Foreshortened femora in fetuin-A-deficient *Ahsg*^*-/-*^ mice indicated a role for fetuin-A in bone formation. We studied early postnatal bone development in fetuin-A-deficient mice and discovered that femora from *Ahsg*^*-/-*^ mice exhibited severely displaced distal epiphyses and deformed growth plates, similar to the human disease slipped capital femoral epiphysis (SCFE). The growth plate slippage occurred in 70% of *Ahsg*^*-/-*^ mice of both sexes around three weeks postnatal. At this time point, mice weaned and rapidly gained weight and mobility. Epiphysis slippage never occurred in wildtype and heterozygous *Ahsg*^*+/-*^ mice. Homozygous fetuin-A-deficient *Ahsg*^*-/-*^ mice and, to a lesser degree, heterozygous *Ahsg*^*+/-*^ mice showed lesions separating the proliferative zone from the hypertrophic zone of the growth plate. The hypertrophic growth plate cartilage in long bones from *Ahsg*^*-/-*^ mice was significantly elongated and V-shaped until three weeks of age and thus prior to the slippage. Genome-wide transcriptome analysis of laser-dissected distal femoral growth plates from 13-day-old *Ahsg*^*-/-*^ mice revealed a JAK-STAT-mediated inflammatory response including a 550-fold induction of the chemokine *Cxcl9*. At this stage, vascularization of the elongated growth plates was impaired, which was visualized by immunofluorescence staining. Thus, fetuin-A-deficient mice may serve as a rodent model of growth plate pathologies including SCFE and inflammatory cartilage degradation.

## Introduction

Endochondral ossification is a tightly controlled developmental sequence of chondrogenic cell differentiation, proliferation and hypertrophy, followed by vascularization and cartilage mineralization. Eventually, calcified cartilage is remodeled into mineralized and vascularized bone [1]. This highly dynamic sequence of events progresses in a confined space–the growth plate–and involves cell layers connected by extracellular matrices with greatly varying mechanical properties. The growth plate architecture supports rapid skeletal growth, but also introduces inherent instability along the boundaries of stacked cells and tissue layers. In order to maintain overall growth plate stability and to prevent the formation of fault lines, the progression from proliferative to hypertrophic cartilage must be tightly controlled. Growth plate fracture or slippage is indeed observed in pre-pubertal fast growing skeletons [2–4]. Remodeling of mineralized cartilage and concurrent vascularization may be particularly critical, because both, mineralization and vascularization, greatly affect the stability of the growth plate. Non-collagen bone proteins are widely believed to regulate bone matrix stability and turnover [5]. Fetuin-A / α_2_-Heremans-Schmid-glycoprotein (gene name *Ahsg*) is one of the most abundant non-collagen proteins in mineralized bone [6]. Fetuin-A is not expressed in bone, but is constitutively secreted by the liver into the circulation. Due to its high affinity towards calcium phosphate [5,7] however, fetuin-A accumulates in mineralized bone and dentin [5,6], suggesting a role for this plasma protein in mineralized bone metabolism [8–14]. Fetuin-A is a highly abundant glycoprotein of 52 kDa containing a cluster of acidic amino acid residues in its amino-terminal cystatin-like protein domain D1 that mediates calcium phosphate binding [10]. In solutions supersaturated with calcium and phosphate, fetuin-A forms colloidal complexes with calcium phosphate termed calciprotein particles, CPPs. The formation of colloidal CPPs facilitates the transport of bulk calcium phosphate in the body [15]. Fetuin-A has not been shown to influence the formation of mineral nuclei but rather shields them to prevent their aggregation and thus mineral precipitation. Accordingly, fetuin-A was found to regulate mineralization in primary rat calvarial osteoblasts, calcifying vascular smooth muscle cells [9,16] and in collagen mineralization assays [17].

Fetuin-A-deficient *Ahsg*^*-/-*^ mice allowed to study *in vivo* the role of fetuin-A in bone metabolism [11,12]. Adult *Ahsg*^*-/-*^ mice displayed shorter femora with increased cortical thickness, while all other bones appeared normal. Femoral growth plates in *Ahsg*^*-/-*^ mice were fragmented and disordered, and unresorbed cartilage islands were present in the metaphyses suggesting a defect in osteochondrogenic development. We conducted these first studies in mice on a mixed genetic background C57BL/6-129/Sv. Later on, we established *Ahsg*^*-/-*^ mice on pure genetic backgrounds C57BL/6 (B6,*Ahsg*^*-/-*^) and DBA/2 (D2,*Ahsg*^*-/-*^) [14,18,19]. In the absence of fetuin-A as a regulator of mineralized matrix metabolism, D2,*Ahsg*^*-/-*^ mice calcified spontaneously in their soft tissues [14], while B6,*Ahsg*^*-/-*^ mice calcified only when challenged with heminephrectomy and a high mineral diet [18,19]. These findings established fetuin-A as a systemic inhibitor of pathological ectopic calcification. Due to renal calcification, D2,*Ahsg*^*-/-*^ mice also developed secondary hyperparathyroidism, therefore showing osteoporosis which complicated their foreshortened femur phenotype. We revisited the role of fetuin-A in bone growth in C57BL/6 mice with a focus on bone mineralization [20]. We confirmed the shorter femur phenotype, but did not observe any differences on the micro-mechanical, micro-structural or cellular level in bone tissue. We hypothesized that the decreased length of the femora was caused by premature growth plate mineralization.

Because long bones develop through endochondral ossification, foreshortened femora in *Ahsg*^*-/-*^ mice indicate a defect in this developmental process. The two previous studies regarding the bone phenotype in *Ahsg*^*-/-*^ mice used adult animals [12,20]. We hypothesized that any developmental defect causing the dysplasia formation in *Ahsg*^*-/-*^ femora should affect early postnatal endochondral ossification. Here, we analyzed postnatal bone development by histology, computed tomography and genome-wide expression analysis of growth plate cartilage to identify gene regulatory networks preceding the developmental defect.

## Materials and methods

### Animals

All animal experiments were conducted in agreement with the recommendations of the Federation for Laboratory Animal Science Associations FELASA, and were approved by the animal welfare committee of the Landesamt für Natur-, Umwelt- und Verbraucherschutz (LANUV) of the state of North Rhine Westphalia (84–02.04.2015.A153). All mice were maintained in a temperature-controlled room on a 12-hour day/night cycle. Food and water were given *ad libitum*. We studied fetuin-A deficient C57BL/6-*Ahsg*^*-/-*^ mice and their heterozygous and wildtype littermates from heterozygous matings [14].

### Measurement of bone growth

Mice were euthanized by cervical dislocation and femora were isolated. Bone length was determined from calibrated photographs (newborn mice) or using a caliper (all other ages) by measuring proximal to distal from the greater trochanter to the outer ends of the condyles. Distal femoral width was determined at the widest point between the two femoral epicondyles.

### Cell proliferation measurement

Cell proliferation was assessed using the Click-iT® Plus EdU Proliferation Kit (Thermo Fisher Scientific). Thirteen-day-old male mice were injected with 50 mg 5-ethynyl-2´-deoxyuridine (EdU) per kg bodyweight (5 mg/ml EdU solution in PBS) and sacrificed after 2 hours. Bones were fixed, decalcified and paraffin-embedded as described below. Paraffin sections were stained according to the manufacturer’s protocol.

### Histology and immunofluorescence

For paraffin histology, bones were fixed in 4% paraformaldehyde (PFA) in PBS for 24 h and subsequently decalcified for up to 10 days in 250 mM ethylenediaminetetraacetic acid (EDTA) in PBS followed by dehydration and paraffin embedding. For morphological analysis, sections were stained with safranin O and fast green FCF. For Diff-Quick histology, a commercially available kit was used (Medion Grifols Diagnostics). Slides were unmasked with citrate buffer prior to antibody staining. The following primary antibodies were used for immunofluorescent staining: rabbit anti-mouse fetuin-A antiserum developed in-house [21], polyclonal rabbit anti-mouse STAT1 IgG (#sc-346, Santa Cruz), monoclonal rabbit anti-mouse pSTAT1 IgG (#7649, Cell Signaling), polyclonal rabbit anti-mouse CD3 (#A0452, Dako). We used Alexa546-goat anti-rabbit IgG antibody (#A-11010, Thermo Fisher) or Alexa488-goat anti-rabbit IgG antibody (#A-11008, Thermo Fisher) as secondary antibody and counterstained with DAPI (4',6-diamidino-2-phenylindole). For immunohistochemistry, we used rat anti-mouse F4/80 IgG antibody (#MCA497, Serotec) and rat anti-mouse CD45 (#553076, BD Pharmigen) and used secondary biotinylated goat anti-rat IgG antibody (#112-065-167, Jackson ImmunoResearch). Antibody binding was visualized with the Vectastain ABC HRP Kit (Vector Laboratories) with 3,3′-Diaminobenzidine (DAB) as substrate. Slides were counterstained with methyl green.

Apoptosis was visualized using the terminal deoxynucleotidyl transferase dUTP nick end labeling (TUNEL) method by staining sections with the DeadEnd™ Fluorometric TUNEL System (Promega) according to the manufacturer’s protocol.

For staining of the vasculature, bones were fixed in zinc fixative for 24 h, decalcified as described above, embedded in OCT (Sakura) and frozen in isopentane cooled with dry ice. Cryosections of 60 μm were stained with rat anti-mouse CD31 IgG (#RM5200, life technologies), and Alexa546-goat anti-rat IgG (#A11081, Thermo Fisher) secondary antibody and counterstained as described above.

### Computed tomography

Femora were fixed as described above and stored in 80% ethanol. Samples were placed in a radiotranslucent sample holder filled with PBS to prevent desiccation. Micro-computed tomography (μCT) scanning was performed using a μCT 40 desktop cone-beam microCT (Scanco Medical) with a voxel size of 10 μm (1000 projections per slice with 2048 samples and 200 s sample time at a tube energy of 55 kVp with an intensity of 145 μA). Reconstructed samples were visualized using Philips Imalytics [22]. Angle measurements were performed using Fiji image analysis software [23].

### Laser capture microdissection and microarray analysis

For microarray analysis, three individual 13-day-old male mice per genotype (*Ahsg*^*+/+*^, *Ahsg*^*+/-*^, *Ahsg*^*-/-*^) were used. From each mouse, the right femur was directly frozen in OCT in isopentane cooled with dry ice. Cryo-section preparation was performed under nuclease-free conditions to maintain RNA integrity. Sections of 14 μm were mounted on PEN-membrane slides (Zeiss). Slides were fixed with 95% ethanol for 2 min, dehydrated with 100% ethanol for 1 min, air dried at room temperature for 5 min and stored at -80°C for up to 48 h until further processing. Laser capture microdissection (LCM) was performed using a Zeiss P.A.L.M. micro laser system. For each sample, approx. 40 frozen sections were prepared and processed. Per section, the entire growth plate (resting zone, proliferative zone and hypertrophic zone) was isolated and captured in an adhesive cap (Zeiss). The isolated tissue was suspended in 50 μl extraction buffer (PicoPure RNA Isolation Kit, Thermo Fisher Scientific), incubated at 42°C for 20 min and stored at -80°C until RNA isolation, which was carried out according to the manufacturer’s protocol.

RNA quality and quantity were assessed using NanoDrop (Thermo Fisher Scientific) and Agilent Bioanalyzer (Agilent Technologies). For microarray analysis, 100 ng RNA per sample were transcribed and labeled with GeneChip WT PLUS Reagent Kit (Affymetrix) according to the manufacturer’s protocol. Labeled cDNA was hybridized to Affymetrix GeneChip Mouse Transcriptome Array 1.0, and visualized with the Affymetrix staining kit. The Affymetrix Expression Console software was utilized for gene data analysis. Data was normalized with the SST-RMA algorithm. Robust mean signal values were calculated using the Tukey’s biweight estimator. For calculating significance (p-value), the non-parametric Wilcoxon’s rank test was used. Probe sets with a p-value <0.05 were considered as differentially expressed.

In order to validate the microarray data, a portion of the RNA used for gene chip analysis was reverse transcribed (Maxima First Strand cDNA Synthesis Kit, Thermo Fisher Scientific) into cDNA, which served as template for quantitative real-time PCR of seven target genes (*Cxcl9*, *Ifit1*, *Usp18*, *Gbp2*, *Igtp*, *Il1b*, *Stat1*), which were among the most highly differentially expressed genes in the microarray analysis. S1 Table shows the corresponding primer sequences. Primers were previously tested for efficiency. Fold changes were determined using the ΔΔCT method and glyceraldehyde 3-phosphate dehydrogenase *Gapdh* as the reference gene. Maxima SYBR Green/ROX qPCR Master Mix (Thermo Scientific) was used for qPCR and 50 ng of cDNA were added per reaction.

### Quantitative PCR Analysis

Growth plates from distal femora and proximal tibiae of thirteen-day-old mice (n = 4 per genotype), were dissected manually under a stereo microscope. Additionally, bone tissue and the remaining epiphyses were collected from the same animals. As control, liver samples were taken from the same *Ahsg*^*+/+*^ animals. RNA was isolated (GeneJET RNA purification Kit, Thermo Fischer) and the subsequent cDNA synthesis and qPCR were carried out as described above. The respective *Ahsg* primer sequences are shown in S1 Table. Fold changes were determined using the ΔΔCT method with *Gapdh* as reference gene and were normalized to *Ahsg*^*+/+*^ liver.

### Cibersort analysis

We used the Bioconductor BiomaRt package to map mouse symbols to human symbols [24]. The expression of all genes with known human symbols was provided to Cibersort [25]. Cibersort estimated the putative proportion of immune cells using a reference set with 22 sorted immune cell subtypes (LM22) for each microarray experiment.

### Statistical analysis

All statistical analyses were carried out using GraphPad Prism version 5.0c for Mac, GraphPad Software, La Jolla, USA. Bone length and width measurement, growth plate zone length measurement as well as qPCR analysis were statistically assessed using One-Way ANOVA combined with Bonferroni’s Multiple Comparison Test. Growth plate angle measurement was statistically assessed using Student’s t-test.

## Results

**Fig 1:**
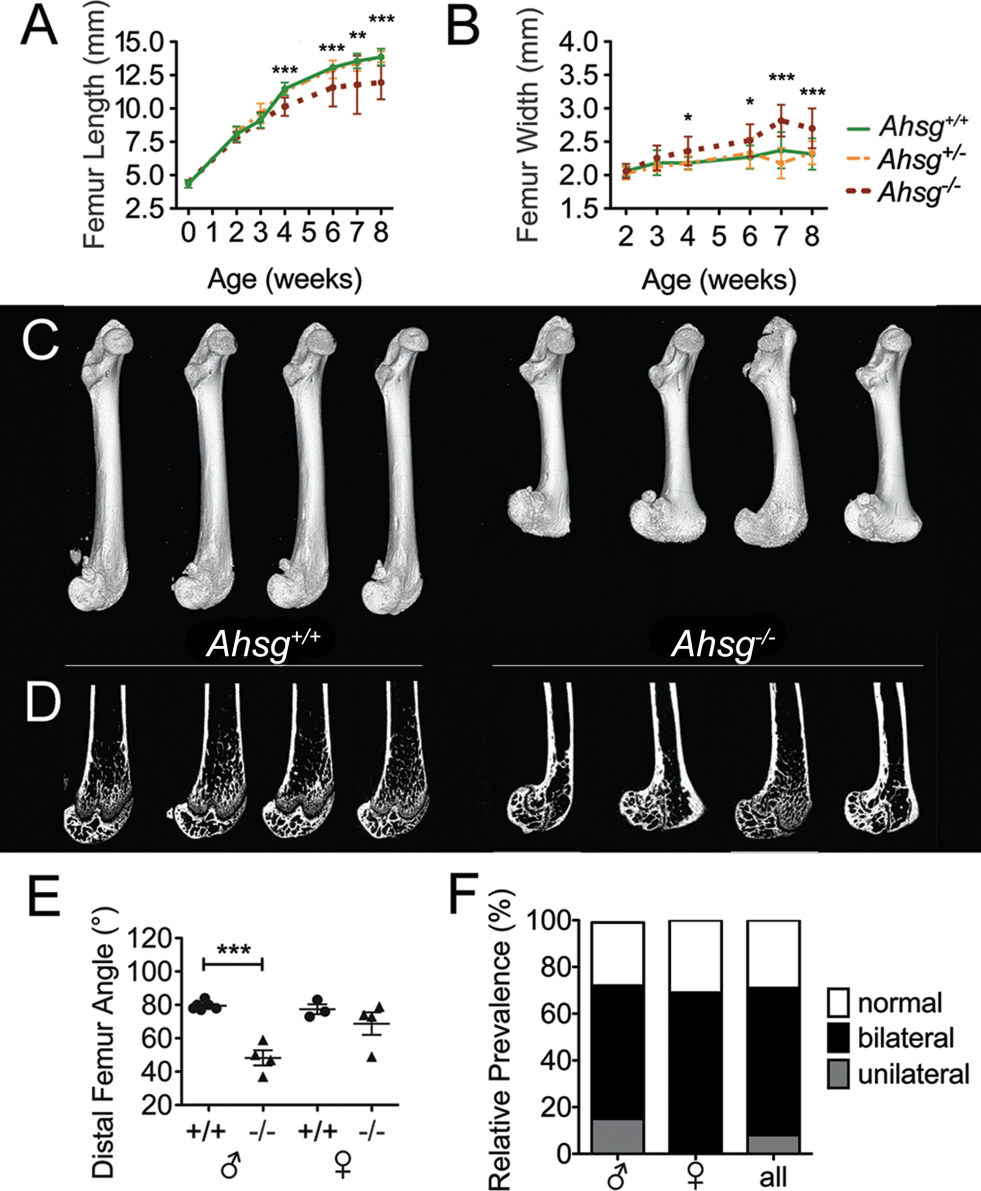
Bone dysplasia in fetuin-A deficient mice. (A) Growth curves of femora from both, male and female *Ahsg*^*+/+*^, *Ahsg*^*+/-*^ and *Ahsg*^*-/-*^ mice reveal that femora of *Ahsg*^*-/-*^ mice were significantly shortened compared to their littermates at the age of four weeks and older. Error bars show SD. Data was analyzed by One-Way ANOVA: *p<0.05, **p<0.01, ***p<0.001. (B) Measurement of distal femur width shows a widening of the distal femoral epiphysis in *Ahsg*^*-/-*^ mice at the age of four weeks and older. (C) Three-dimensional reconstructions from μCT measurements of eight-week-old male mice and (D) matching 2D μCT cross-sections. Micro-CT analysis shows a posterior rotation of the distal femoral epiphysis in *Ahsg*^*-/-*^ mice. (E) The angle of the distal femoral epiphysis relative to the shaft (see S1 Fig) was significantly decreased in male *Ahsg*^*-/-*^ mice. Error bars show SD. Data was analyzed by Student’s t-test: ***p<0.001. (F) Prevalence of the dysplasia in male and female C57BL/6 *Ahsg*^*-/-*^ mice. Mice at the age of four weeks and older were evaluated for dysplasia by either bone length measurements from isolated bones or from radiographic images. Out of 62 *Ahsg*^*-/-*^ mice, 44 showed dysplasia in their distal femur in one or both legs, 18 mice had no obvious phenotype anomaly.

For each genotype (*Ahsg*^*+/+*^, *Ahsg*^*+/-*^, *Ahsg*^*-/-*^) and age, the length of femora was measured in at least ten mice of both sexes (Fig 1A, for sample numbers see S2 Table). While femoral growth was similar in wildtype and *Ahsg*^*+/-*^ mice, femora from *Ahsg*^*-/-*^ mice were significantly shorter from four weeks of age onwards, indicating a decisive event before the age of four weeks. Fig 1B shows an increased width of the distal femoral epiphysis in *Ahsg*^*-/-*^ mice starting from four weeks of age, concurrent with their shortening. Femora from *Ahsg*^*-/-*^ mice exhibited malformations in the distal femur compared to femora from *Ahsg*^*+/+*^ and *Ahsg*^*+/-*^ mice. In order to investigate the morphological anomalies in *Ahsg*^*-/-*^ femora in more detail, bones from eight-week-old mice were studied using micro-computed tomography (μCT). The three-dimensional visualization revealed a notable feature that had gone unnoticed in all previous studies on the bone phenotype of *Ahsg*^*-/-*^ mice: the distal femoral epiphysis was rotated posterior, the lateral and medial condyles and epicondyles were flattened and widened, which resulted in narrowing of the intercondylar fossa (Fig 1C). The corresponding 2D cross-sections (Fig 1D) also showed a severe deformation of the epiphyseal plates in *Ahsg*^*-/-*^ femora. The rotation of the distal femoral epiphysis was quantified measuring the angle of the distal femoral growth plate relative to the shaft as shown in S1 Fig. This angle was significantly decreased in male *Ahsg*^*-/-*^ mice (Fig 1E). The prevalence of the dysplasia was nonetheless similar in male and female mice (Fig 1F). In total, 44 out of 62 *Ahsg*^*-/-*^ mice had distal femur dysplasia in one or both legs and 18 mice had no obvious phenotype anomaly. Taken together, the prevalence of the dysplasia was around 70% for both sexes.

**Fig 2:**
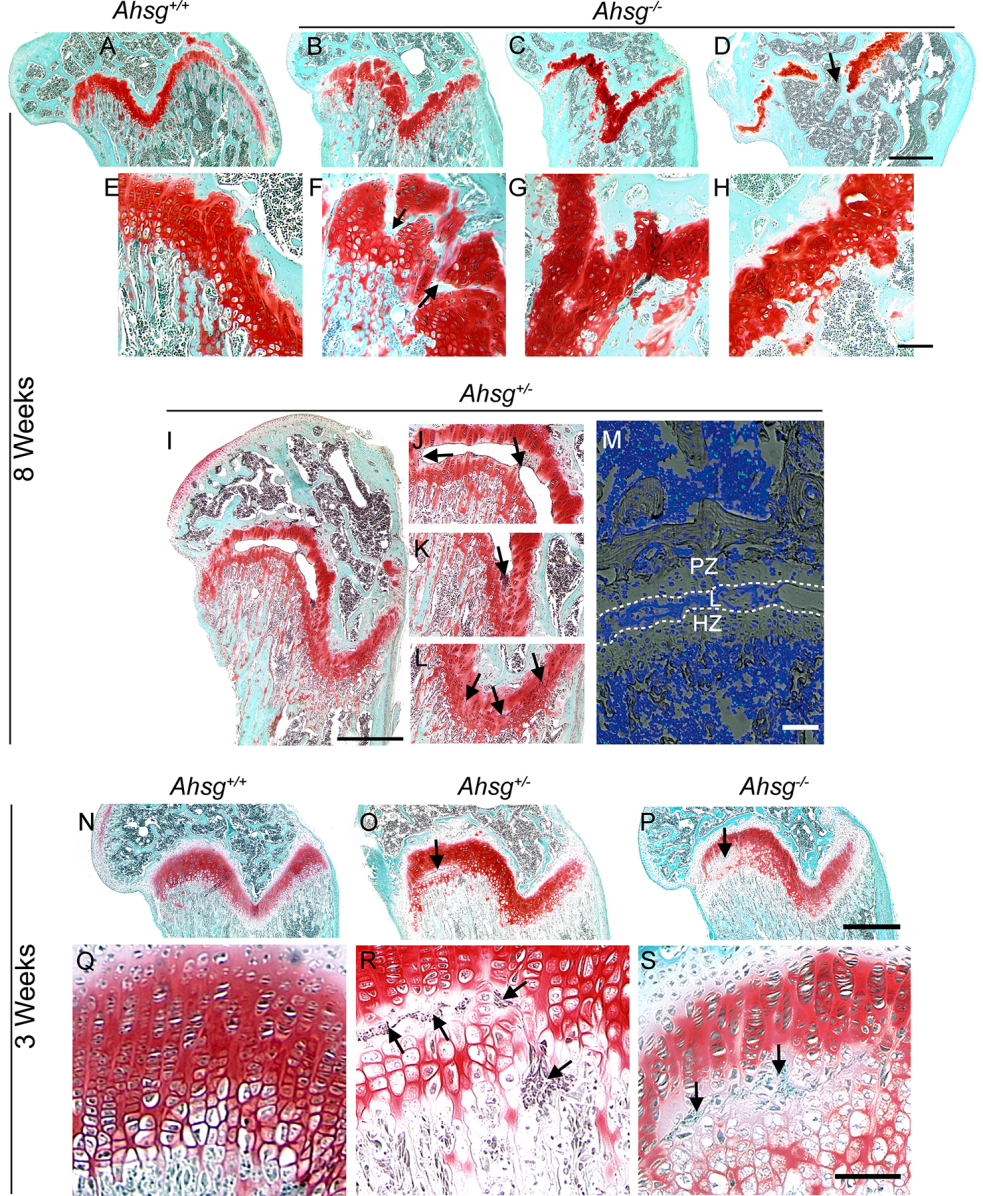
Growth plate histology. Paraffin sections of decalcified bone were stained with safranin O and fast green. (A-H) Sections of distal femora from eight-week-old *Ahsg*^*-/-*^ mice show growth plate anomalies. (A-D) show entire growth plates (scale bar 500 μm), while (E-H) shows higher magnifications (scale bar 200 μm) of each corresponding growth plate above. (I-L) Bone section of an eight-week-old heterozygous *Ahsg*^*+/-*^ mouse showing an extended lesion spanning half the width of the growth plate (scale bar 500 μm). (J-L) show details of I with arrows pointing to lesion-associated cells. (M) TUNEL staining of an *Ahsg*^*+/-*^ growth plate. (PZ, proliferative zone; L, lesion; HZ, hypertrophic zone; scale bar 100 μm). Cells in the lesion stained TUNEL-negative, while few cells in the bone marrow were TUNEL-positive (green). (N-S) Sections of distal femora from three-week-old mice. (N-P) show entire growth plates (scale bar 500 μm), while (Q-S) show higher magnifications (scale bar 200 μm) of each corresponding growth plate above. Lesions with lesion-associated cells were never found in wildtype *Ahsg*^*+/+*^ growth plates, but were present in both heterozygous *Ahsg*^*+/-*^ (O, R) and in homozygous fetuin-A deficient *Ahsg*^*-/-*^ (P, S) growth plates (arrows). Diminished safranin O staining around lesion-associated cells indicated cartilage matrix degradation.

We analyzed histological sections from femora of eight-week-old (Fig 2A–2M) and three-week-old mice (Fig 2N–2S). Growth plate pathology in eight-week-old fetuin-A-deficient mice varied widely. Compared to wildtype mice (Fig 2A and 2E), growth plate chondrocytes in *Ahsg*^*-/-*^ mice (Fig 2B–2D and 2F–2H) appeared disordered and had lost their zonal arrangement. Occasionally, bony lesions disrupted the growth plate (Fig 2F, arrows) or the growth plate was partially closed (Fig 2D, arrow).

In heterozygous mice, five out of 14 *Ahsg*^*+/-*^ mice analyzed had inconspicuous distal femoral growth plates, which were indistinguishable from those of *Ahsg*^*+/+*^ mice. Nine mice had however, at least one lesion in one or both of their distal femoral growth plates. Fig 2I shows a histological section of a particularly large lesion lined with lesion-associated cells. Fig 2M shows a negative terminal deoxynucleotidyl transferase dUTP nick end labeling (TUNEL) staining in these cells, ruling out that the lesion-lining cells were apoptotic chondrocytes.

The deformation of the epiphysis around the growth plate and the disorganization of the growth plate morphology in *Ahsg*^*-/-*^ mice were similar to the proximal epiphysis slippage occurring in the human disorder slipped capital femoral epiphysis (SCFE). To study the development of the growth plate slippage in fetuin-A-deficient mice, we histologically examined femora from younger mice. Fig 2N–2S show femora from three-week-old mice. Already at three weeks postnatal, lesions separating the proliferative zone from the hypertrophic zone and lesion-associated cells were present in the distal femoral growth plates of both, *Ahsg*^*-/-*^ and *Ahsg*^*+/-*^ mice, but never in wildtype mice. Safranin O staining of the region around these cells appeared less intense, indicating cartilage degradation. Panels A-E in S2 Fig illustrate that the lesion-associated cells in growth plates of four-week-old *Ahsg*^*-/-*^ and eight-week-old *Ahsg*^*+/-*^ mice stained negative for cell surface markers CD45, F4/80 and CD3, while cells in the bone marrow of the same bone sections stained positive for these cell surface markers (panels F-J in S2 Fig), suggesting that the lesion-associated cells were neither hematopoietic cells, macrophages, nor T cells. Diff-Quick histology of lesion-associated cells from four-week-old and eight-week-old growth plate lesions (panels K and L in S2 Fig) were compared to blood smears from wildtype mice (panels M-O in S2 Fig). Lesions at both ages had only mononuclear cells ruling out the presence of osteoclasts and granulocytes (panel M in S2 Fig). Thus the lesion-associated cells were morphologically similar to leukocytes, albeit CD45, F4/80, CD3 triple negative.

Macroscopically, femora appeared normal up until three weeks postnatal with no signs of condyle slippage. Thus epiphyseal slippage and ultimately distal femoral dysplasia occurred at or shortly after three weeks postnatal. This is the time of weaning, when the mice rapidly gain weight and mobility. We suggest that the increased biomechanical load of destabilized growth plates associated with increased body weight and mobility likely caused the haphazard slippage of growth plates in 70% of *Ahsg*^*-/-*^ mice.

**Fig 3:**
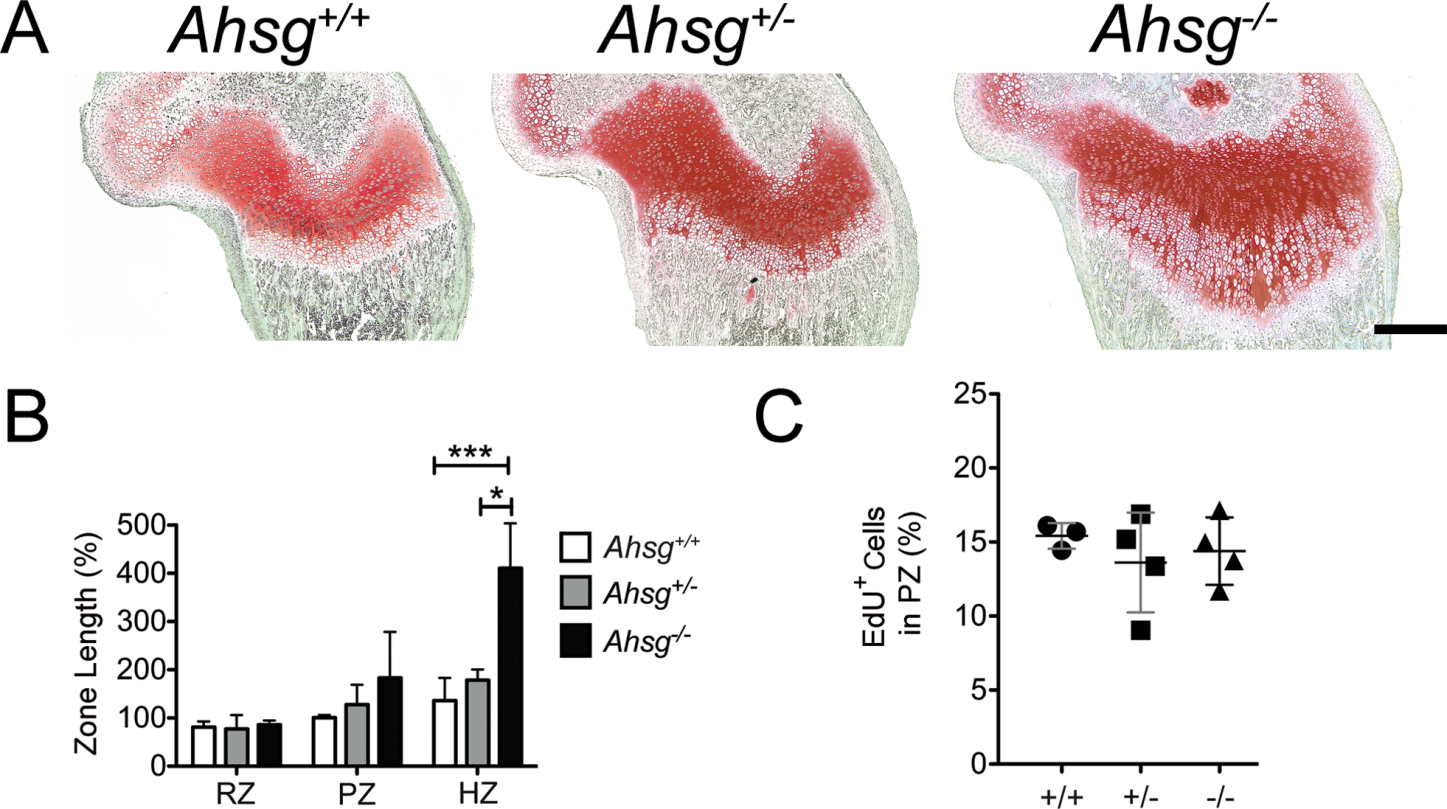
Growth plate morphology in 13-day-old mice. (A) Histological staining with safranin O/fast green revealed an elongated hypertrophic zone in 13-day-old *Ahsg*^*-/-*^ mice. Scale bar is 500 μm. (B) Length measurements of reserve (RZ), proliferative (PZ) and hypertrophic zones (HZ) from 13-day-old male *Ahsg*^*+/+*^, *Ahsg*^*+/-*^ and *Ahsg*^*-/-*^ mice were performed on paraffin sections. Error bars show SD. Data was analyzed by One-Way ANOVA: *p<0.05, ***p<0.001. (C) The percentage of EdU^+^ cells indicated that proliferation was essentially identical in the proliferative zone of 13-day-old mice of all genotypes.

In newborn mice up until about three weeks of age, hypertrophic zones in the growth plates of *Ahsg*^*-/-*^ mice were markedly elongated and V-shaped. Fig 3 shows representative sections from distal femora of 13-day-old mice and the corresponding zone length measurements. S3 Fig shows that tibia, humerus and ulna also had elongated hypertrophic zones, indicating that the developmental defect occurred in all long bones.

We asked whether the elongation was caused by an increase in proliferation. We labeled proliferating cells by injecting intraperitoneally 5-ethynyl-2'-deoxyuridine (EdU) into 13-day-old mice, and determined the percentage of EdU positive chondrocytes in the proliferative zone in distal femora two hours after injection. The percentage of EdU positive cells was identical for all genotypes (Fig 3C, S4 Fig), indicating that the growth plate elongation was not caused by increased proliferation.

**Fig 4:**
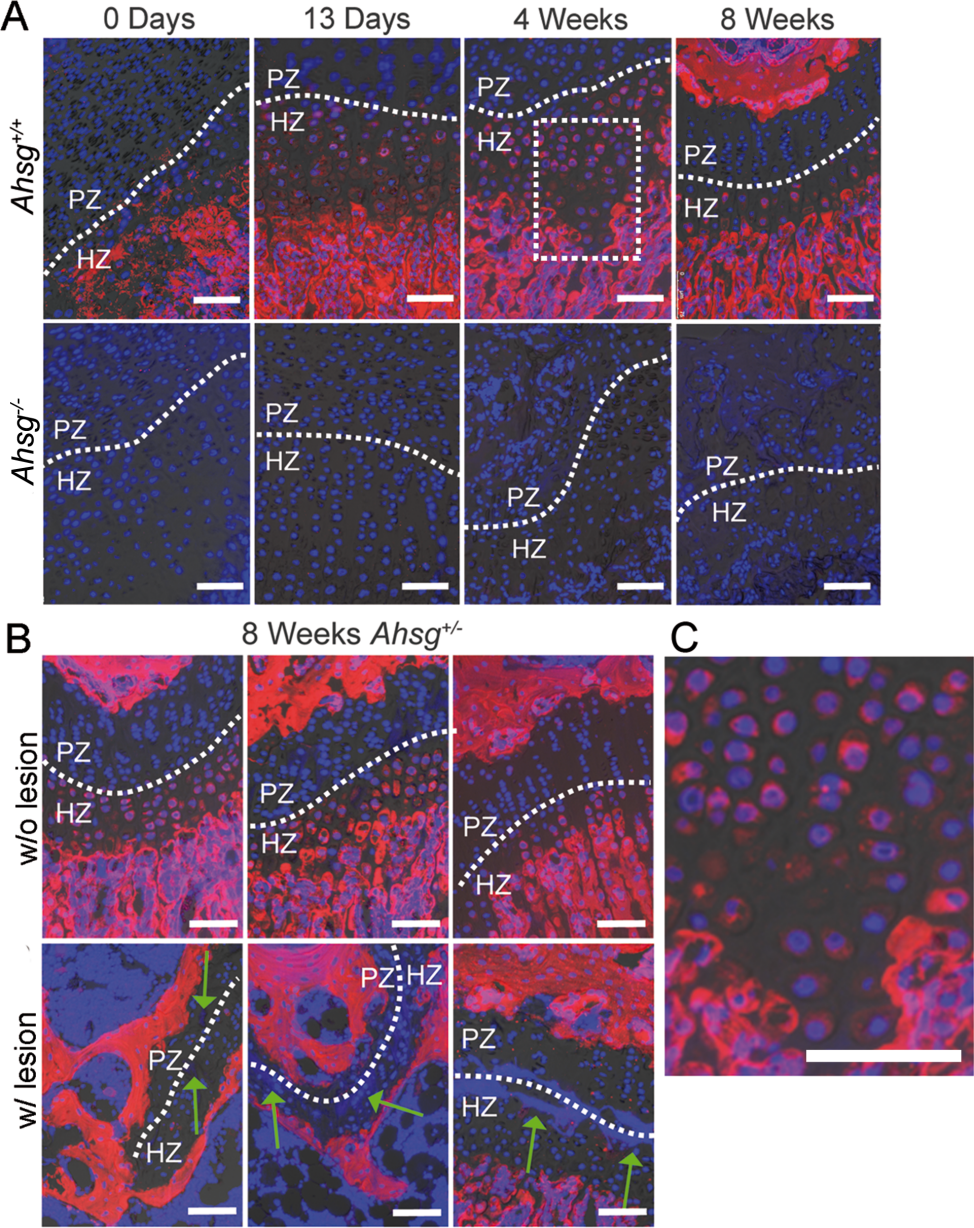
Growth plate histology and immunofluorescent localization of fetuin-A in distal femoral growth plates. (A) Decalcified femur paraffin sections from mice of different ages were stained with an anti-fetuin-A antibody (red) and nuclei were counterstained with DAPI (blue). In the growth plate, fetuin-A was localized in hypertrophic chondrocytes. *Ahsg*^*-/-*^ mice served as negative control (lower panel). (B) Similar to wildtype mice, fetuin-A was detected in hypertrophic chondrocytes from eight-week-old *Ahsg*^*+/-*^ mice. Fetuin-A staining was negative in growth plates containing a lesion (green arrows). (C) A magnified view of the marked area in (A) shows the cytoplasmic localization of fetuin-A in hypertrophic chondrocytes. Scale bars are 75 μm.

In view of the drastic growth plate phenotype in *Ahsg*^*-/-*^ mice, we analyzed, whether fetuin-A protein was at all present in the growth plate, even though bone and cartilage do not normally express fetuin-A mRNA. To this end, we determined the distribution of fetuin-A protein in growth plate cartilage by fluorescent antibody staining of decalcified paraffin sections. The hypertrophic zones of distal femoral growth plates (Fig 4A) stained positive for fetuin-A. In contrast to the strong matrix staining of mineralized bone, hypertrophic chondrocytes stained positive for fetuin-A predominantly in their cytoplasm (Fig 4A and 4C). Growth plates from eight-week-old *Ahsg*^*+/-*^ mice without lesions exhibited the same staining pattern as growth plates from *Ahsg*^*+/+*^ mice (Fig 4B upper panel, Fig 4C). In contrast, fetuin-A staining was negative in the growth plate of *Ahsg*^*+/-*^ mice which exhibited a lesion (Fig 4B lower panel), suggesting that lesions were strictly associated with the absence of fetuin-A protein. We performed qRT-PCR analysis of excised growth plate cartilage from 13-day-old mice to detect low-level *Ahsg* mRNA expression (S5 Fig). We detected roughly 10,000-fold lower *Ahsg* mRNA expression in growth plates from *Ahsg*^+/+^ and *Ahsg*^+/-^ mice compared to liver of wildtype mice. Similar results were obtained for bone and epiphysis tissue, corroborating an earlier expression study [26]. Thus, our data suggest low expression of *Ahsg* mRNA in growth plate chondrocytes compared to liver hepatocytes.

In order to identify gene regulatory networks involved in growth plate lesion formation, we analyzed the gene expression profiles of laser-dissected growth plate chondrocytes from *Ahsg*^*+/+*^, *Ahsg*^*+/-*^ and *Ahsg*^*-/-*^ mice. We selected growth plate cartilage from mice 13 days of age, a time point at which lesions had not yet formed. Whole distal femoral growth plates were isolated from three mice per genotype using laser capture microdissection. High quality RNA was isolated and further processed for whole genome DNA microarray analysis. The microarray data are accessible at NCBI Gene Expression Omnibus through GEO Series accession number GSE105139 [27].

**Fig 5:**
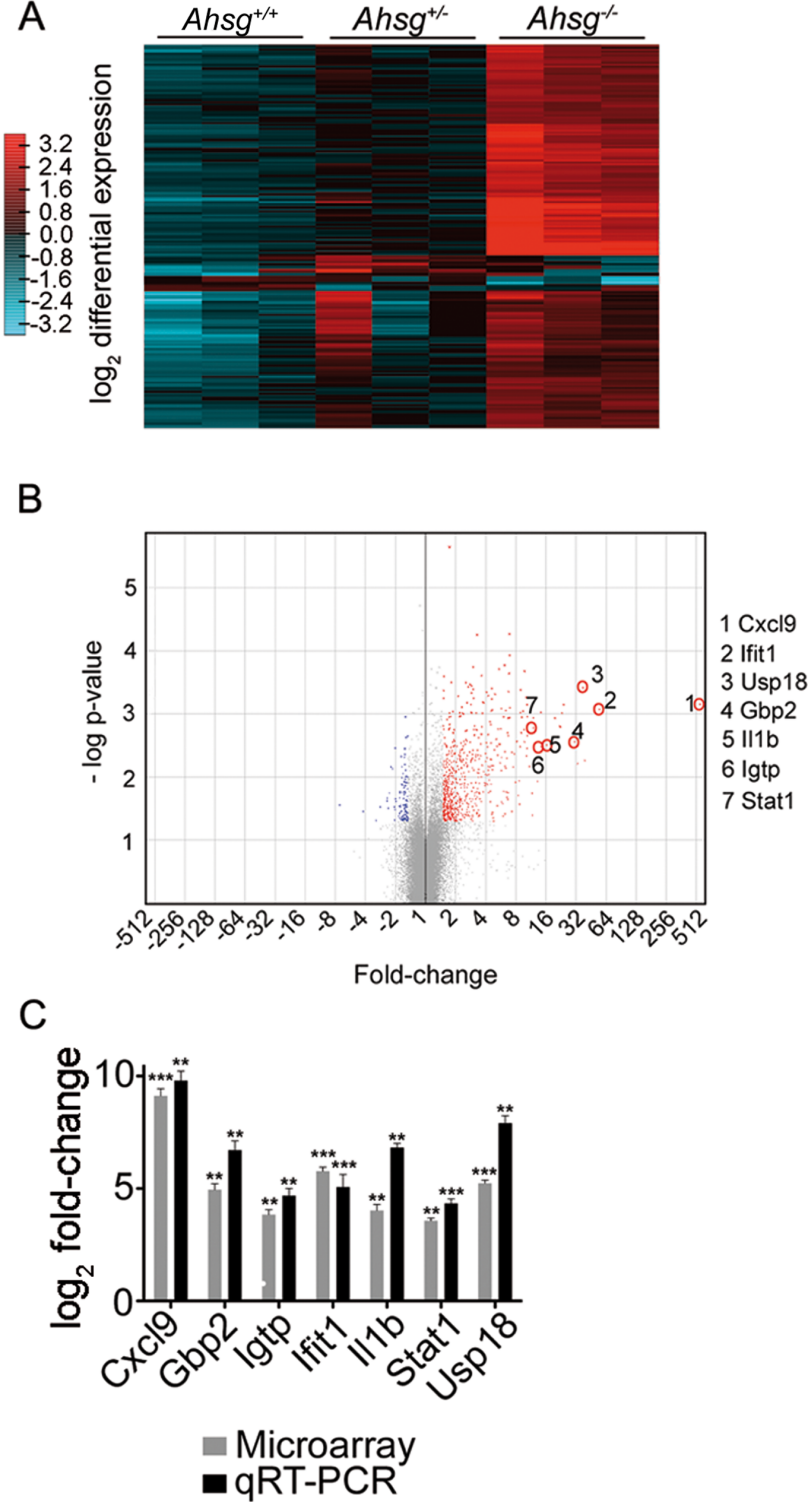
Genome-wide gene expression analysis reveals the induction of pro-inflammatory genes in growth plates of 13-day-old *Ahsg*^*-/-*^ mice. (A) Heatmap representation of the normalized gene expression in individual growth plates shows consistently strong induction of large gene clusters in fetuin-A deficient *Ahsg*^*-/-*^ mice compared to wildtype *Ahsg*^*+/+*^ mice. Gene expression in growth plates of *Ahsg*^*+/-*^ was heterogeneous and had partial overlap with both *Ahsg*^*-/-*^ and *Ahsg*^*+/+*^ genotypes. (B) Volcano plot comparing the normalized growth plate gene expression between *Ahsg*^*-/-*^ and *Ahsg*^*+/+*^ mice. The plot shows that more significantly differentially expressed genes were induced (red), and few genes were significantly repressed (blue). Genes marked with red circles were used for validation of microarray data with qRT-PCR. (C) The seven most highly differentially induced genes were validated using qRT-PCR. The graph represents the log_2_ fold changes in expression in *Ahsg*^*-/-*^ compared to *Ahsg*^*+/+*^ samples. Data was analyzed using Student’s t-test: **p<0.005, ***p<0.001.

Fig 5A shows a heatmap representation of differential gene expression of growth plate chondrocytes from *Ahsg*^*+/+*^, *Ahsg*^*+/-*^ and *Ahsg*^*-/-*^ mice. Clusters of differentially expressed genes were identified with high statistical significance when comparing *Ahsg*^*-/-*^ and *Ahsg*^*+/+*^ samples. While expression data in each three biological replicates from either *Ahsg*^*-/-*^ or *Ahsg*^*+/+*^ mice were essentially identical, *Ahsg*^*+/-*^ mice showed partial overlap with both *Ahsg*^*-/-*^ and *Ahsg*^*+/+*^ genotypes, precluding a meaningful comparison.

Gene chip expression analysis of laser-dissected individual growth plates and of liver tissue corroborated the results of qRT-PCR performed on excised growth plates and liver tissue, in that fetuin-A mRNA expression was high in wildtype liver (mean spot intensity log_2_ = 14.03), but more than 700-fold lower (mean spot intensity log_2_ = 4.52) in growth plate cartilage (S3 Table). The mRNA expression value for growth plate cartilage was in fact numerically lower than *Ahsg*^*-/-*^ liver (mean spot intensity log_2_ = 5.21), which has no *Ahsg* gene and therefore cannot possibly express fetuin-A mRNA. Collectively the data on fetuin-A expression suggest that bone tissue in general and growth plate cartilage in particular express very low fetuin-A mRNA that might nevertheless have contributed to the fetuin-A protein detected in hypertrophic chondrocytes by immunofluorescence.

As illustrated by the volcano plot, more genes were induced than repressed in the growth plates of *Ahsg*^*-/-*^ mice compared to *Ahsg*^*+/+*^ mice (Fig 5B). The repressed genes were mostly pseudogenes and the highest repression was 3.13-fold in the predicted gene *Gm24683*. Therefore we focused our analysis on the induced genes. In order to validate the microarray data we selected seven genes which were strongly induced in *Ahsg*^*-/-*^ growth plates and performed qRT-PCR using the original RNA from the microarray analysis as template. Strong gene induction was confirmed for all candidate genes (Fig 5C).

All genes with an induction of at least 1.25-fold were included in a gene enrichment analysis using the databases Gene Ontology and WikiPathways [28,29]. The results showed a high enrichment of inflammatory gene regulatory pathways. The majority of the most highly significantly induced genes in *Ahsg*^*-/-*^ growth plates mapped to the type II interferon (IFNɣ) signaling pathway [WikiPathways WP1253][29].

IFNɣ is mainly produced by natural killer (NK) cells and T cells [30]. We probed for the presence of NK and T cell mRNA in our gene expression data sets using the bioinformatics tool Cibersort [25] (S4 Table). Cibersort assumes the gene expression data to be derived from hematopoietic cells, and estimates the fraction of each type of immune cell for each sample by comparing the gene expression data to the signatures of different immune cell subsets. As the gene expression data originated from growth plate chondrocytes, the number of hematopoietic cells is naturally zero. Nevertheless, we employed Cibersort to probe for immune cells that may be present in growth plates of the various genotypes. We determined an immune cell signature compatible with non-activated macrophages (M0), with no differences between genotypes. Importantly, Cibersort analysis did not suggest the presence of NK cell or T cell signatures in the expressed genome derived from growth plates of either *Ahsg*^*+/+*^, *Ahsg*^*+/-*^ or *Ahsg*^*-/-*^ mice. The fact that both immunostaining and Cibersort were negative for T cells suggests that the interferon type gene regulatory network is growth plate-intrinsic, and may be a salient part of growth plate physiology. This interferon type gene regulatory network was strongly induced in chondrocytes in fetuin-A-deficient mice.

**Fig 6:**
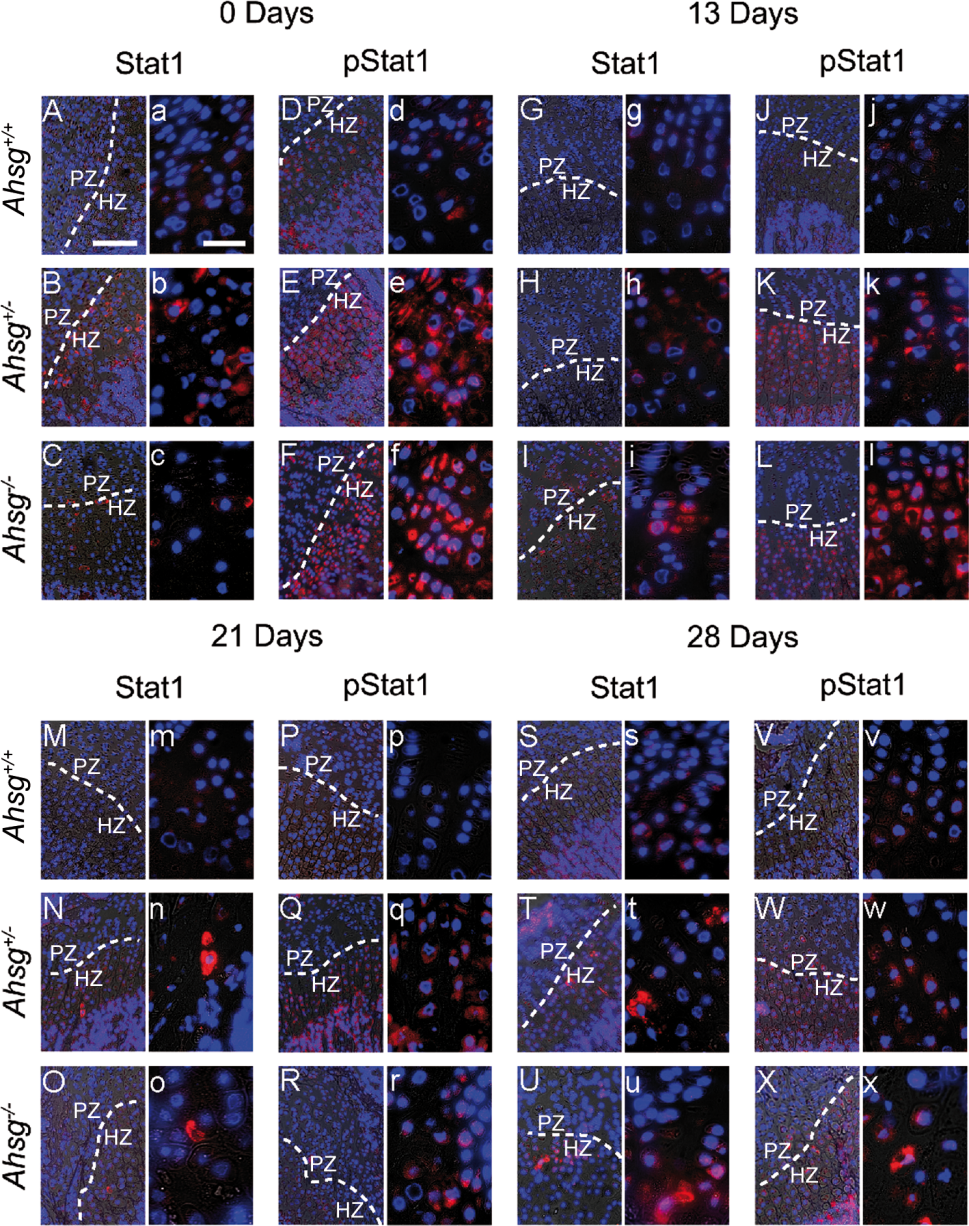
Immunofluorescent localization of STAT1 and phospho-STAT1 (pSTAT1) in distal femoral growth plates. Decalcified paraffin sections were stained with antibodies for STAT1 and pSTAT1 (red) and nuclei were counterstained with DAPI (blue). The figure shows representative micrographs for each time point and each genotype recorded with a 20-fold (uppercase letter) and a 63-fold lens (lowercase letter). STAT1 and pSTAT1 signal was mainly localized in the hypertrophic zone. The signal was increased in *Ahsg*^*+/-*^ and *Ahsg*^*-/-*^ mice of all ages. Scale bars are 150 μm for micrographs taken at lower and 20 μm for micrographs taken at a higher magnification.

Furthermore, we investigated, whether interferon-triggered signaling was active in growth plate chondrocytes. The microarray analysis showed a twelve-fold induction of signal transducer and activator of transcription 1 (STAT1) in *Ahsg*^*-/-*^ growth plates. The regulation of STAT1 expression occurs through a positive feedback loop and its up-regulation strongly indicates the activation of the JAK-STAT signaling pathway [31]. Using antibody staining, we detected STAT1 and phospho-STAT1 (pSTAT1) protein in growth plates of *Ahsg*^*+/+*^, *Ahsg*^*+/-*^ and *Ahsg*^*-/-*^ mice of different ages (Fig 6). Hypertrophic chondrocytes of newborn *Ahsg*^*+/+*^ mice stained positive for STAT1 and pSTAT1 suggesting active JAK-STAT signaling in physiological bone growth. Hypertrophic and, to a lesser degree, proliferative growth plate chondrocytes from both, *Ahsg*^*+/-*^ and *Ahsg*^*-/-*^ mice, generally exhibited a stronger staining for STAT1 and pSTAT1 compared to *Ahsg*^*+/+*^ mice at all examined time points. Staining was strongest in newborn mouse growth plates.

**Fig 7:**
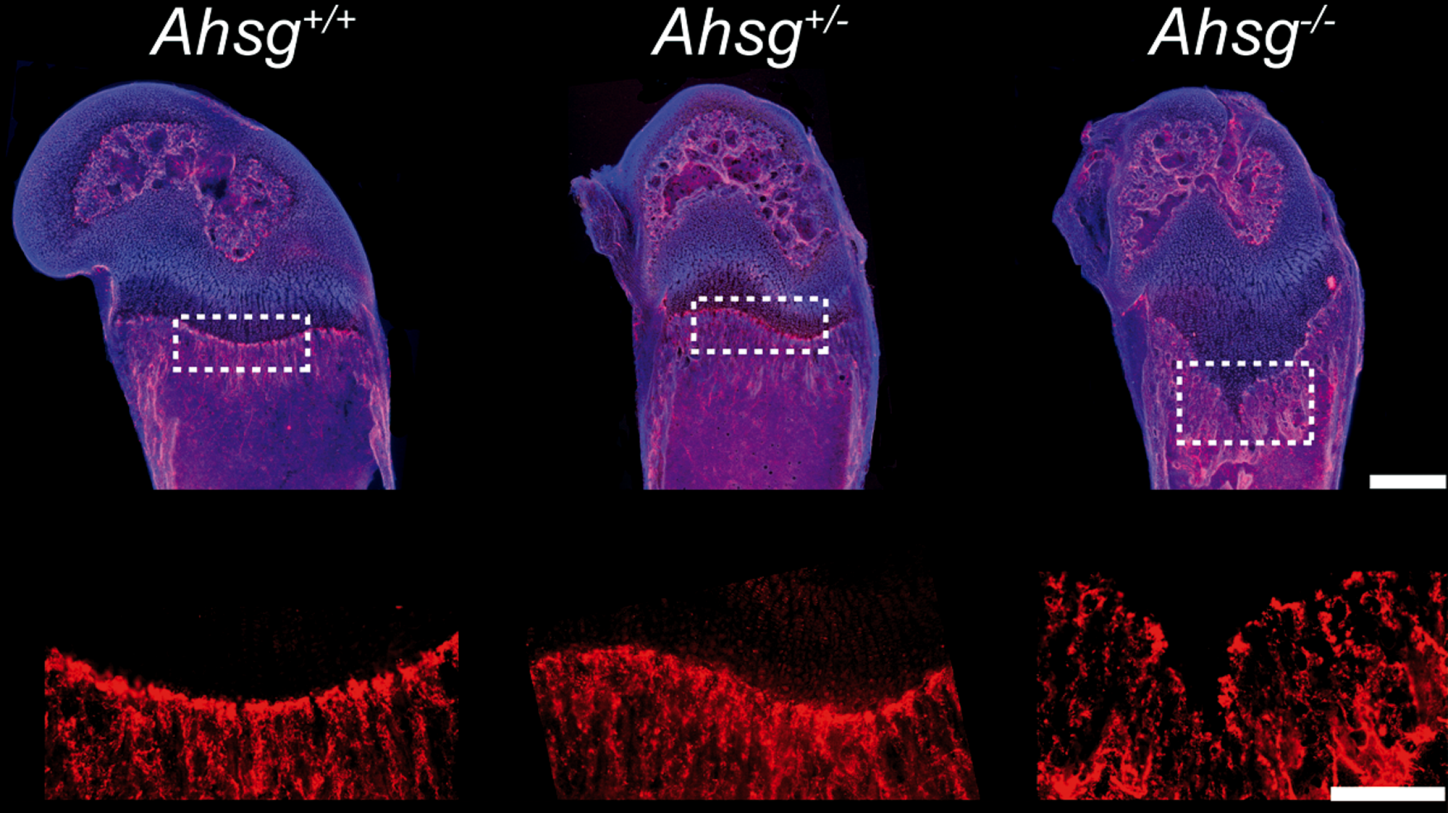
Immunofluorescent localization of the vasculature in distal femoral growth plates. Thick cryo-sections of femora from 13-day-old mice were stained with anti-CD31 antibody (red) and nuclei were counterstained with DAPI (blue) to visualize the vascular network. We examined 5 *Ahsg*^*+/+*^ samples, 6 *Ahsg*^*+/-*^ samples and 8 *Ahsg*^*-/-*^ samples. Upper panel shows overviews representing overlays of blue, red and bright-field channels. Lower panel shows a magnified view of the red channel (CD31). Fewer capillary loops reaching the chondro-osseous junction were found in *Ahsg*^*-/-*^ mice compared to *Ahsg*^*+/+*^ and *Ahsg*^*+/-*^ littermates. Scale bar upper panel is 500 μm, scale bar lower panel is 250 μm.

The chemokine *CXC-Ligand 9* (*Cxcl9*) was the most highly induced gene in the growth plates of *Ahsg*^*-/-*^ mice, with an induction of 558-fold. CXCL9 was recently identified to counteract VEGF in bone vascularization, but has never been reported in growth plate cartilage [32]. As we found a strong induction of *Cxcl9* in growth plates of 13-day-old *Ahsg*^*-/-*^ mice, the elongated hypertrophic zones might be a consequence of deficient growth plate vascularization. Therefore we stained the vasculature in 13-day-old mice on thick histological sections. Fig 7 illustrates that the number of capillary loops at the chondro-osseous junction in distal femoral growth plates from *Ahsg*^*-/-*^ mice was indeed reduced compared to *Ahsg*^*+/+*^ mice, suggesting a role for CXCL9 in the initial stages of the growth plate dysplasia in *Ahsg*^*-/-*^ mice.

## Discussion

Fetuin-A is a major non-collagen protein in bone, but its exact role in bone physiology is unclear. Fetuin-A-deficient *Ahsg*^*-/-*^ mice have shortened femoral bones, indicating a crucial role for fetuin-A in endochondral ossification.

Here, we studied the role of fetuin-A in endochondral ossification by investigating femur development and gene expression in growth plate chondrocytes. Computed tomography enabled a 3-dimensional and detailed visualization of the foreshortened femora from *Ahsg*^*-/-*^ mice. We noticed that the distal femoral epiphysis and the growth plate were both severely deformed, indicating a slippage of the growth plate during endochondral ossification, a phenotype similar to the human disease slipped capital femoral epiphysis (SCFE).

In *Ahsg*^*-/-*^ distal femoral growth plates, we found an unusually strong induction of inflammatory genes, especially genes regulated by IFNɣ. *Cxcl9* was the most strongly induced (558-fold) gene in *Ahsg*^*-/-*^ growth plates. The chemokine CXCL9 is expressed during inflammation and promotes the chemotaxis of pro-inflammatory cells, especially T_h_1 lymphocytes [33,34]. Therefore, we probed for the presence of IFNɣ producing cells including NK cells and especially T cells. The bioinformatic data analysis suggested that NK cells and T cells were not present in *Ahsg*^*-/-*^, *Ahsg*^*+/-*^ and *Ahsg*^*+/+*^ growth plates. Furthermore, we confirmed increased STAT1 expression and phosphorylation in *Ahsg*^*-/-*^ and *Ahsg*^*+/-*^ growth plate chondrocytes compared to wildtype. Taken together, our results suggest that JAK-STAT signaling is growth plate chondrocyte-intrinsic, especially during the hypertrophic state. In the absence of fetuin-A, this physiological JAK-STAT interferon type gene regulatory network is strongly induced resulting in e.g. the 558-fold induction of *Cxcl9*. We hypothesize that hypertrophic chondrocyte-derived CXCL9 may contribute to physiological cartilage remodeling by recruiting remodeling cells. Furthermore, diminished calcified matrix metabolism in the absence of fetuin-A may lead to an exaggerated *Cxcl9* induction causing excessive remodeling cell activation and lesion formation in the growth plate instead of physiological remodeling.

Our results showed that until about three weeks postnatal, growth plates from *Ahsg*^*-/-*^ mice had elongated V-shaped hypertrophic zones. A recent publication demonstrated that the chemokine CXCL9 inhibits angiogenesis through direct antagonization of VEGF [32]. Staining of the bone vasculature showed that the vascularization at the chondro-osseous junction in *Ahsg*^*-/-*^ mice was indeed diminished. Thus, the elongated hypertrophic zone in *Ahsg*^*-/-*^ mice most likely resulted from a lack of vascular invasion caused by excessive *Cxcl9* expression in the growth plate. Similarly, mouse models with a deficiency in either cartilage remodeling or vascular invasion (MMP13-, MMP9-, VEGF-deficient mice) have likewise elongated hypertrophic zones, attesting to the close interplay of vascularization and tissue remodeling during endochondral ossification [35–37].

Both, *Ahsg*^*-/-*^ and *Ahsg*^*+/-*^ mice showed lesion-associated cells in their growth plates, located between the proliferative and the hypertrophic zone at three weeks of age. Decreased safranin O staining indicated cartilage degradation around these cells. It is presently unclear, if these cells represent chondrocytic cells degrading matrix, or if they represent infiltrating bone marrow cells causing the lesion.

Immunofluorescent staining demonstrated that fetuin-A was predominantly localized in the cytoplasm of hypertrophic chondrocytes. Consistently, qRT-PCR showed a very low level of *Ahsg* expression in growth plates. These data suggest that, in addition to the fetuin-A protein released by the liver, the fetuin-A protein expressed locally by hypertrophic chondrocytes might also contribute to growth plate development. Therefore, deletion of *Ahsg* in hypertrophic chondrocytes might partially contribute to the growth plate defects observed in fetuin-A-deficient mice. Further studies involving liver- or growth plate-specific deletion of *Ahsg* may allow the dissection of the roles of fetuin-A derived from each source in the normal growth plate development.

The slippage of the distal femoral growth plate in *Ahsg*^*-/-*^ mice is comparable to the human disease slipped capital femoral epiphysis (SCFE) [3], which features a similar slippage of the growth plate, however affecting the proximal femoral epiphysis. We attribute the difference in location to differences in anatomy and thus in mechanical loading of the proximal and distal epiphysis in mice and men. We propose that fetuin-A-deficient mice may serve as a model for SCFE.

SCFE is the most common hip disorder in adolescents, with an incidence of 0.2 to 10 per 100,000 per year [38,39]. Biomechanical, endocrine, and biochemical factors have all been quoted to play a role in the SCFE etiology. SCFE patient material is rare and available only after the slippage has occurred, making it difficult to investigate triggering events. A recent study on SCFE patient material has demonstrated the induction of immune system response genes in the growth plates of SCFE patients [38]. The authors interpreted the finding as a secondary effect due to mechanical damage. Since we observed a pro-inflammatory interferon type gene regulatory network in growth plates from *Ahsg*^*-/-*^ mice well before any lesions had formed, we suggest that inflammatory tissue remodeling may in fact trigger the mechanical weakening and slippage of the growth plate in SCFE patients.

Fetuin-A itself may be involved in SCFE development. Heterozygous *Ahsg*^*+/-*^ mice which produce about half the amount of fetuin-A compared to their *Ahsg*^*+/+*^ littermates also had lesions in their growth plates. Fetuin-A is a negative acute phase protein, thus its blood levels markedly decrease upon systemic inflammation [40]. Therefore, endochondral ossification may become disturbed during systemic inflammation in childhood causing depressed fetuin-A blood levels.

Our results suggest that the absence of fetuin-A elicits an inflammatory response in growth plate chondrocytes. The exact molecular events leading to the expression of IFNɣ-responsive genes in the growth plate and the subsequent changes in gene expression, growth plate slippage and dysplasia will be the subject of further studies.

## Supporting information

S1 Fig Measurement of growth plate angle relative to the shaft. Angles were measured on contrast-inverted 2D sagittal cross-sections from μCT measurements of bones from eight-week-old *Ahsg*^*+/+*^ and *Ahsg*^*-/-*^ mice as shown in this figure.
(TIF)

S2 Fig Characterization of lesion-associated cells. Histological sections were prepared from paraffin-embedded decalcified bone samples. (A-E) illustrate lesion-associated cells in growth plates of four-week-old *Ahsg*^*-/-*^ and eight-week-old *Ahsg*^*+/-*^ mice staining negative for cell surface markers CD45, F4/80 and CD3. (F-J) show bone marrow cells of the same bone sections staining positive for CD45, F4/80 and CD3, suggesting that the lesion-associated cells were neither hematopoietic cells, macrophages, or T cells. (K-O) Diff-Quick histology of lesion-associated cells in growth plate lesions from four-week-old and eight-week-old mice (K, L) were compared to blood smears from wildtype mice (M-O), suggesting that lesions at both ages had only mononuclear cells ruling out the presence of osteoclasts and granulocytes.
(TIF)

S3 Fig *Ahsg*^*-/-*^ mice have elongated and V-shaped hypertrophic zones in their long bones. Safranin O/fast green staining of decalcified paraffin sections from 13-day-old mice show elongated and V-shaped hypertrophic zones in the growth plates of long bones.
(TIF)

S4 Fig Representative histological femoral sections used for proliferation measurement. Mice were injected with EdU, sacrificed after 2 h and EdU (seen as green nuclei) was visualized on decalcified histological sections.
(TIF)

S5 Fig Quantitative PCR of *Ahsg* mRNA expression in growth plate cartilage, bone and epiphyses normalized to *Ahsg*^*+/+*^ liver. Growth plates, adjacent bone tissue and the remaining epiphyses from distal femora and proximal tibiae from 13-day-old *Ahsg*^*+/+*^, *Ahsg*^*+/-*^ and *Ahsg*^*-/-*^ mice, were manually dissected (n = 4 mice per group). Additionally, *Ahsg*^*+/+*^ liver samples were taken (n = 4). RNA was isolated, reverse transcribed and *Ahsg* mRNA expression was analyzed using qRT-PCR. Fold-changes were determined using the ΔΔCt method, using *Ahsg*^*+/+*^ liver as control. Expression values were compared to liver expression values using One-Way ANOVA: ***p<0.001. N.D. = not detectable.
(TIF)

S1 Table Primer sequences used for quantitative real-time PCR. Primers were designed using NCBI primer-BLAST, or taken from Primer Bank. We ensured that all primers were spanning exon-exon junctions and primed the coding region of the gene.
(DOCX)

S2 Table Number of bones used for bone length measurements. (DOCX)

S3 Table Fetuin-A mRNA expression in growth plates. Gene expression is given as the log_2_ of the mean raw spot intensity after normalizing the microarray expression data. The raw spot intensities were averaged over biological replicates. Values for *Ahsg*^*+/+*^ and *Ahsg*^*-/-*^ liver expression were taken from an independent microarray analysis using the same platform. Note that the spot intensities for growth plates of all genotypes and for *Ahsg*^*-/-*^ liver were similarly low at around 2^5^, while the wildtype liver spot intensity was 2^14^.
(DOCX)

S4 Table Cibersort analysis detects no T cells in growth plates of 13-day-old mice. Cibersort assumes that the gene expression data is derived from hematopoietic cells and estimates the fraction of each type of immune cell for each sample by comparing the gene expression data to established signatures of different immune cell subsets. The fractional value for 22 types of immune cells including seven T cell subtypes is listed in each row, thus for each the values add up to 1. Cibersort indicated that the fractions of T cell subtypes in the growth plates were uniformly low with no differences in growth plates derived from *Ahsg*^*+/+*^, *Ahsg*^*+/-*^ and *Ahsg*^*-/-*^ mice. Pearson correlation coefficient, root mean square deviation (MSD) and p-value confirmed the validity of the analysis.
(DOCX)

## References

[1] Kronenberg HM. Developmental regulation of the growth plate. Nature. 2003;423(6937):332–6. doi: 10.1038/nature01657 12748651 10.1038/nature01657

[2] Peterson HA. Epiphyseal growth plate fractures Berlin: Springer-Verlag; 2007.

[3] Peck K, Herrera-Soto J. Slipped capital femoral epiphysis: What’s new? Orthop Clin North Am. 2014;45(1):77–86. doi: 10.1016/j.ocl.2013.09.002 24267209 10.1016/j.ocl.2013.09.002

[4] Packialakshmi B, Rath NC, Huff WE, Huff GR. Poultry femoral head separation and necrosis: A review. Avian Dis. 2015;59(3):349–54. doi: 10.1637/11082-040715-Review.1 26478152 10.1637/11082-040715-Review.1

[5] Termine JD. Non-collagen proteins in bone. Ciba Found Symp. 1988;136:178–202. 3068009 10.1002/9780470513637.ch12

[6] Dickson IR, Poole AR, Veis A. Localisation of plasma α2HS glycoprotein in mineralising human bone. Nature. 1975;256(5516):430–2. 49853 10.1038/256430a0

[7] Triffitt JT, Gebauer U, Ashton BA, Owen ME, Reynolds JJ. Origin of plasma α2HS-glycoprotein and its accumulation in bone. Nature. 1976;262(5565):226–7. 934340 10.1038/262226a0

[8] Jahnen-Dechent W, Heiss A, Schäfer C, Ketteler M. Fetuin-A regulation of calcified matrix metabolism. Circ Res. 2011;108(12):1494–509. doi: 10.1161/CIRCRESAHA.110.234260 21659653 10.1161/CIRCRESAHA.110.234260

[9] Schinke T, Amendt C, Trindl A, Pöschke O, Müller-Esterl W, Jahnen-Dechent W. The serum protein α2-HS glycoprotein/fetuin inhibits apatite formation in vitro and in mineralizing calvaria cells. A possible role in mineralization and calcium homeostasis. J Biol Chem. 1996;271(34):20789–96. 8702833 10.1074/jbc.271.34.20789

[10] Heiss A, DuChesne A, Denecke B, Grötzinger J, Yamamoto K, Renneé T, et al Structural basis of calcification inhibition by α2-HS glycoprotein/fetuin-A: Formation of colloidal calciprotein particles. J Biol Chem. 2003;278(15):13333–41. doi: 10.1074/jbc.M210868200 12556469 10.1074/jbc.M210868200

[11] Jahnen-Dechent W, Schinke T, Trindl A, Müller-Esterl W, Sablitzky F, Kaiser S, et al Cloning and targeted deletion of the mouse fetuin gene. J Biol Chem. 1997;272(50):31496–503. 9395485 10.1074/jbc.272.50.31496

[12] Szweras M, Liu D, Partridge EA, Pawling J, Sukhu B, Clokie C, et al α2-HS glycoprotein/fetuin, a transforming growth factor β/bone morphogenetic protein antagonist, regulates postnatal bone growth and remodeling. J Biol Chem. 2002;277(22):19991–7. doi: 10.1074/jbc.M112234200 11901155 10.1074/jbc.M112234200

[13] Heiss A, Eckert T, Aretz A, Richtering W, Van Dorp W, Schäfer C, et al Hierarchical role of fetuin-A and acidic serum proteins in the formation and stabilization of calcium phosphate particles. J Biol Chem. 2008;283(21):14815–25. doi: 10.1074/jbc.M709938200 18364352 10.1074/jbc.M709938200

[14] Schäfer C, Heiss A, Westenfeld R, Ketteler M, Floege J, Müller-Esterl W, et al The serum protein alpha 2-Heremans-Schmid-glycoprotein/fetuin-A is a systemically acting inhibitor of ectopic calcification. J Clin Invest. 2003;112(3):357–66. doi: 10.1172/JCI17202 12897203 10.1172/JCI17202 PMC166290

[15] Herrmann M, Schäfer C, Heiss A, Gräber S, Kinkeldey A, Büscher A, et al Clearance of fetuin-A-containing calciprotein particles is mediated by scavenger receptor-A. Circ Res. 2012;111(5):575–84. doi: 10.1161/CIRCRESAHA.111.261479 22753077 10.1161/CIRCRESAHA.111.261479

[16] Reynolds JL, Joannides AJ, Skepper JN, McNair R, Schurgers LJ, Proudfoot D, et al Human vascular smooth muscle cells undergo vesicle-mediated calcification in response to changes in extracellular calcium and phosphate concentrations: A potential mechanism for accelerated vascular calcification in ESRD. J Am Soc Nephrol. 2004;15(11):2857–67. doi: 10.1097/01.ASN.0000141960.01035.28 15504939 10.1097/01.ASN.0000141960.01035.28

[17] Nudelman F, Pieterse K, George A, Bomans PHH, Friedrich H, Brylka LJ, et al The role of collagen in bone apatite formation in the presence of hydroxyapatite nucleation inhibitors. Nat Mater. 2010;9(12):1004–9. doi: 10.1038/nmat2875 20972429 10.1038/nmat2875 PMC3084378

[18] Westenfeld R, Schäfer C, Smeets R, Brandenburg VM, Floege J, Ketteler M, et al Fetuin-A (AHSG) prevents extraosseous calcification induced by uraemia and phosphate challenge in mice. Nephrol Dial Transplant. 2007;22(6):1537–46. doi: 10.1093/ndt/gfm094 17389622 10.1093/ndt/gfm094

[19] Westenfeld R, Schäfer C, Krüger T, Haarmann C, Schurgers LJ, Reutelingsperger C, et al Fetuin-A protects against atherosclerotic calcification in CKD. J Am Soc Nephrol. 2009;20(6):1264–74. doi: 10.1681/ASN.2008060572 19389852 10.1681/ASN.2008060572 PMC2689898

[20] Seto J, Busse B, Gupta HS, Schäfer C, Krauss S, Dunlop JWC, et al Accelerated growth plate mineralization and foreshortened proximal limb bones in fetuin-A knockout mice. PLoS One. 2012;7(10):e47338 doi: 10.1371/journal.pone.0047338 23091616 10.1371/journal.pone.0047338 PMC3473050

[21] Denecke B, Gräber S, Schäfer C, Heiss A, Wöltje M, Jahnen-Dechent W. Tissue distribution and activity testing suggest a similar but not identical function of fetuin-B and fetuin-A. Biochem J. 2003;376(1):135–45. 12943536 10.1042/BJ20030676 PMC1223762

[22] Gremse F, Doleschel D, Zafarnia S, Babler A, Jahnen-Dechent W, Lammers T, et al Hybrid μCT-FMT imaging and image analysis. J Vis Exp. 2015;(100):e52770 doi: 10.3791/52770 26066033 10.3791/52770 PMC4512251

[23] Schindelin J, Arganda-Carreras I, Frise E, Kaynig V, Longair M, Pietzsch T, et al Fiji: an open-source platform for biological-image analysis. Nat Methods. 2012;9(7):676–82. doi: 10.1038/nmeth.2019 22743772 10.1038/nmeth.2019 PMC3855844

[24] Durinck S, Moreau Y, Kasprzyk A, Davis S, De Moor B, Brazma A, et al BioMart and Bioconductor: a powerful link between biological databases and microarray data analysis. Bioinformatics. 2005;21(16):3439–40. doi: 10.1093/bioinformatics/bti525 16082012 10.1093/bioinformatics/bti525

[25] Newman AM, Liu CL, Green MR, Gentles AJ, Feng W, Xu Y, et al Robust enumeration of cell subsets from tissue expression profiles. Nat Methods. 2015;12(5):453–7. doi: 10.1038/nmeth.3337 25822800 10.1038/nmeth.3337 PMC4739640

[26] Jahnen-Dechent W, Brylka L, Schinke T, McKee MD. Letter to the Editor, concerning: “FGF23-regulated production of fetuin-A (AHSG) in osteocytes.” Bone. 2016;93. 10.1016/j.bone.2016.01.031 27260648

[27] Edgar R, Domrachev M, Lash AE. Gene Expression Omnibus: NCBI gene expression and hybridization array data repository. Nucleic Acids Res. 2002;30(1):207–10. 11752295 10.1093/nar/30.1.207 PMC99122

[28] Ashburner M, Ball CA, Blake JA, Botstein D, Butler H, Cherry JM, et al Gene Ontology: Tool for the unification of biology. Nat Genet. 2000;25(1):25–9. doi: 10.1038/75556 10802651 10.1038/75556 PMC3037419

[29] Kutmon M, Riutta A, Nunes N, Hanspers K, Willighagen EL, Bohler A, et al WikiPathways: Capturing the full diversity of pathway knowledge. Nucleic Acids Res. 2016;44(D1):D488–94. doi: 10.1093/nar/gkv1024 26481357 10.1093/nar/gkv1024 PMC4702772

[30] Rusinova I, Forster S, Yu S, Kannan A, Masse M, Cumming H, et al INTERFEROME v2.0: An updated database of annotated interferon-regulated genes. Nucleic Acids Res. 2013;41(D1):D1040–6. 23203888 10.1093/nar/gks1215 PMC3531205

[31] Yuasa K, Hijikata T. Distal regulatory element of the STAT1 gene potentially mediates positive feedback control of STAT1 expression. Genes to Cells. 2016;21(1):25–40. doi: 10.1111/gtc.12316 26592235 10.1111/gtc.12316

[32] Huang B, Wang W, Li Q, Wang Z, Yan B, Zhang Z, et al Osteoblasts secrete Cxcl9 to regulate angiogenesis in bone. Nat Commun. 2016;7:13885 doi: 10.1038/ncomms13885 27966526 10.1038/ncomms13885 PMC5171795

[33] Groom JR, Luster AD. CXCR3 in T cell function. Exp Cell Res. 2011;317(5):620–31. doi: 10.1016/j.yexcr.2010.12.017 21376175 10.1016/j.yexcr.2010.12.017 PMC3065205

[34] Karin N, Wildbaum G, Thelen M. Biased signaling pathways via CXCR3 control the development and function of CD4+ T cell subsets. J Leukoc Biol. 2016;99(6):857–62. doi: 10.1189/jlb.2MR0915-441R 26657511 10.1189/jlb.2MR0915-441R

[35] Stickens D. Altered endochondral bone development in matrix metalloproteinase 13-deficient mice. Development. 2004;131(23):5883–95. doi: 10.1242/dev.01461 15539485 10.1242/dev.01461 PMC2771178

[36] Vu TH, Shipley JM, Bergers G, Berger JE, Helms JA, Hanahan D, et al MMP-9/gelatinase B is a key regulator of growth plate angiogenesis and apoptosis of hypetrophic chondrocytes. Cell. 1998;93(3):411–22. 9590175 10.1016/s0092-8674(00)81169-1 PMC2839071

[37] Maes C, Carmeliet P, Moermans K. Impaired angiogenesis and endochondral bone formation in mice lacking the vascular endothelial growth factor isoforms VEGF 164 and VEGF. Mech Dev. 2002;111(2002):61–73. 11804779 10.1016/s0925-4773(01)00601-3

[38] Johnson JS, Weiner DS, Jacquet R, Adamczyk MJ, Morscher MA, Landis WJ. Microarray analysis of slipped capital femoral epiphysis growth plates. J Pediatr Endocrinol Metab. 2016;29(8):971–8. doi: 10.1515/jpem-2016-0023 27390878 10.1515/jpem-2016-0023

[39] Scharschmidt T, Jacquet R, Weiner D, Lowder E, Schrickel T, Landis WJ. Gene expression in slipped capital femoral epiphysis. J Bone Jt Surg. 2009;91(2):366–77. 10.2106/JBJS.G.00039 19181981

[40] Lebreton JP, Joisel F, Raoult JP, Lannuzel B, Rogez JP, Humbert G. Serum concentration of human α2 HS glycoprotein during the inflammatory process: evidence that α2 HS glycoprotein is a negative acute-phase reactant. J Clin Invest. 1979;64(4):1118–29. doi: 10.1172/JCI109551 90057 10.1172/JCI109551 PMC372224

