# Supplementary figures and images for: Post-weaning epiphysiolysis causes distal femur dysplasia and foreshortened hindlimbs in fetuin-A-deficient mice
Source: PLoS One. 2017 Oct 31;12(10):e0187030. doi: 10.1371/journal.pone.0187030 (PMC5663435; doi:10.1371/journal.pone.0187030)

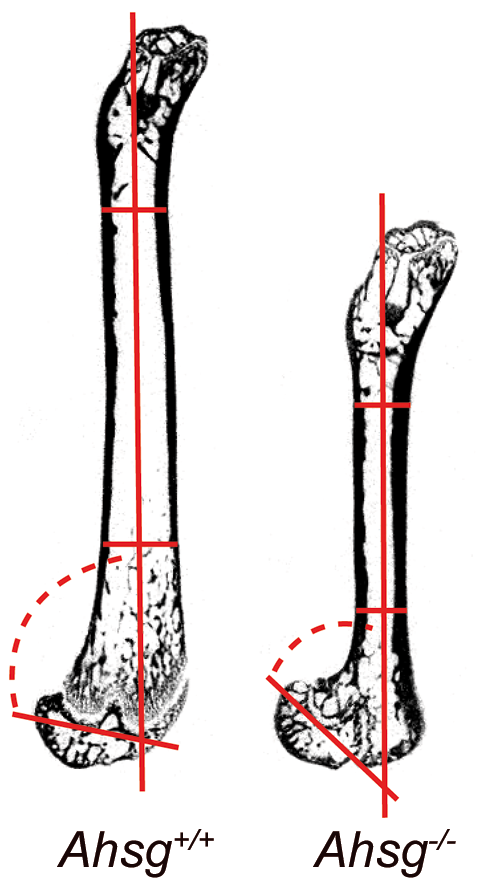

Supplement: S1 Fig — Angles were measured on contrast-inverted 2D sagittal cross-sections from μCT measurements of bones from eight-week-old Ahsg+/+ and Ahsg-/- mice as shown in this figure. (TIF) [file pone.0187030.s001.tif]

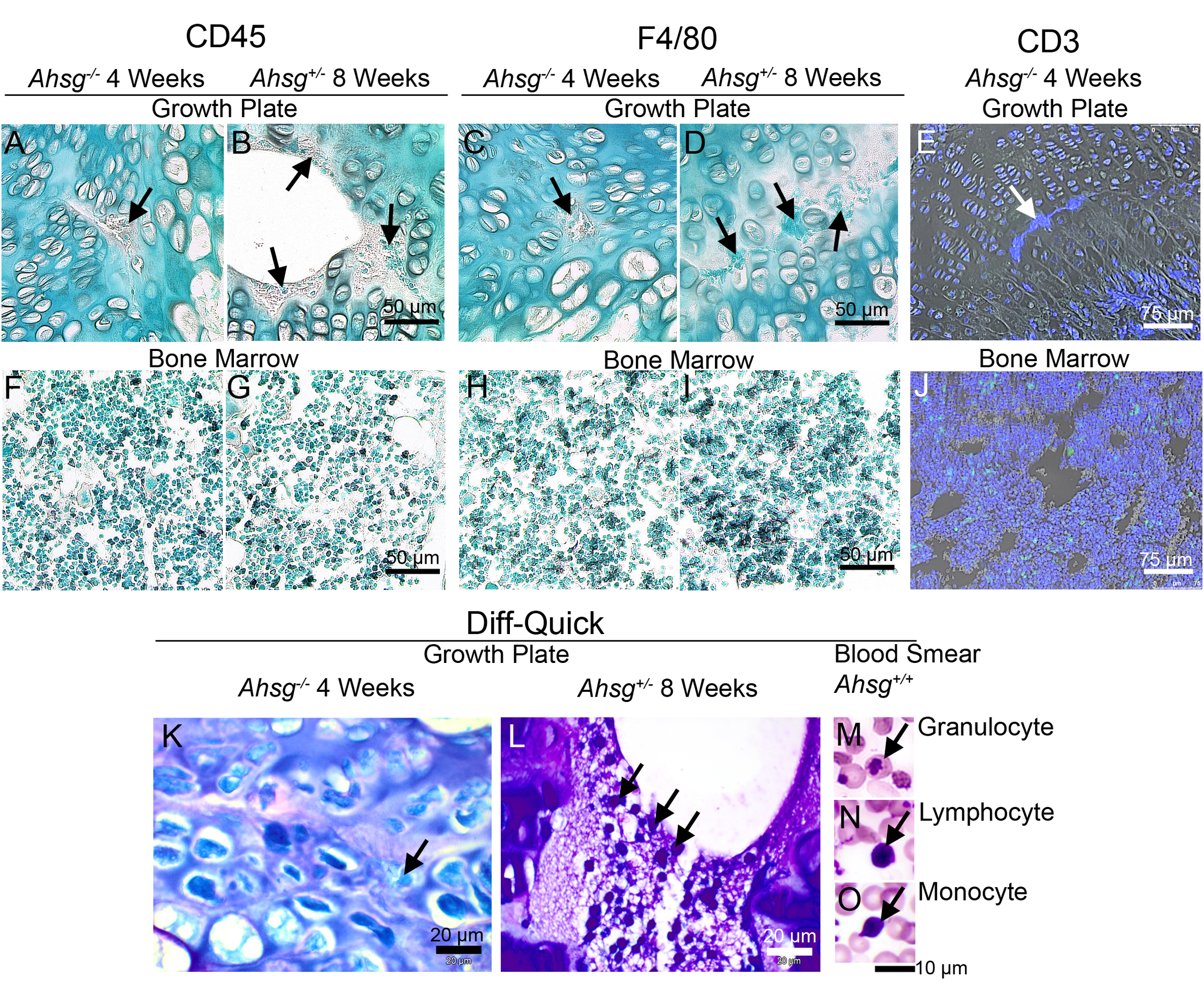

Supplement: S2 Fig — Histological sections were prepared from paraffin-embedded decalcified bone samples. (A-E) illustrate lesion-associated cells in growth plates of four-week-old Ahsg-/- and eight-week-old Ahsg+/- mice staining negative for cell surface markers CD45, F4/80 and CD3. (F-J) show bone marrow cells of the same bone sections staining positive for CD45, F4/80 and CD3, suggesting that the lesion-associated cells were neither hematopoietic cells, macrophages, or T cells. (K-O) Diff-Quick histology of lesion-associated cells in growth plate lesions from four-week-old and eight-week-old mice (K, L) were compared to blood smears from wildtype mice (M-O), suggesting that lesions at both ages had only mononuclear cells ruling out the presence of osteoclasts and granulocytes. (TIF) [file pone.0187030.s002.tif]

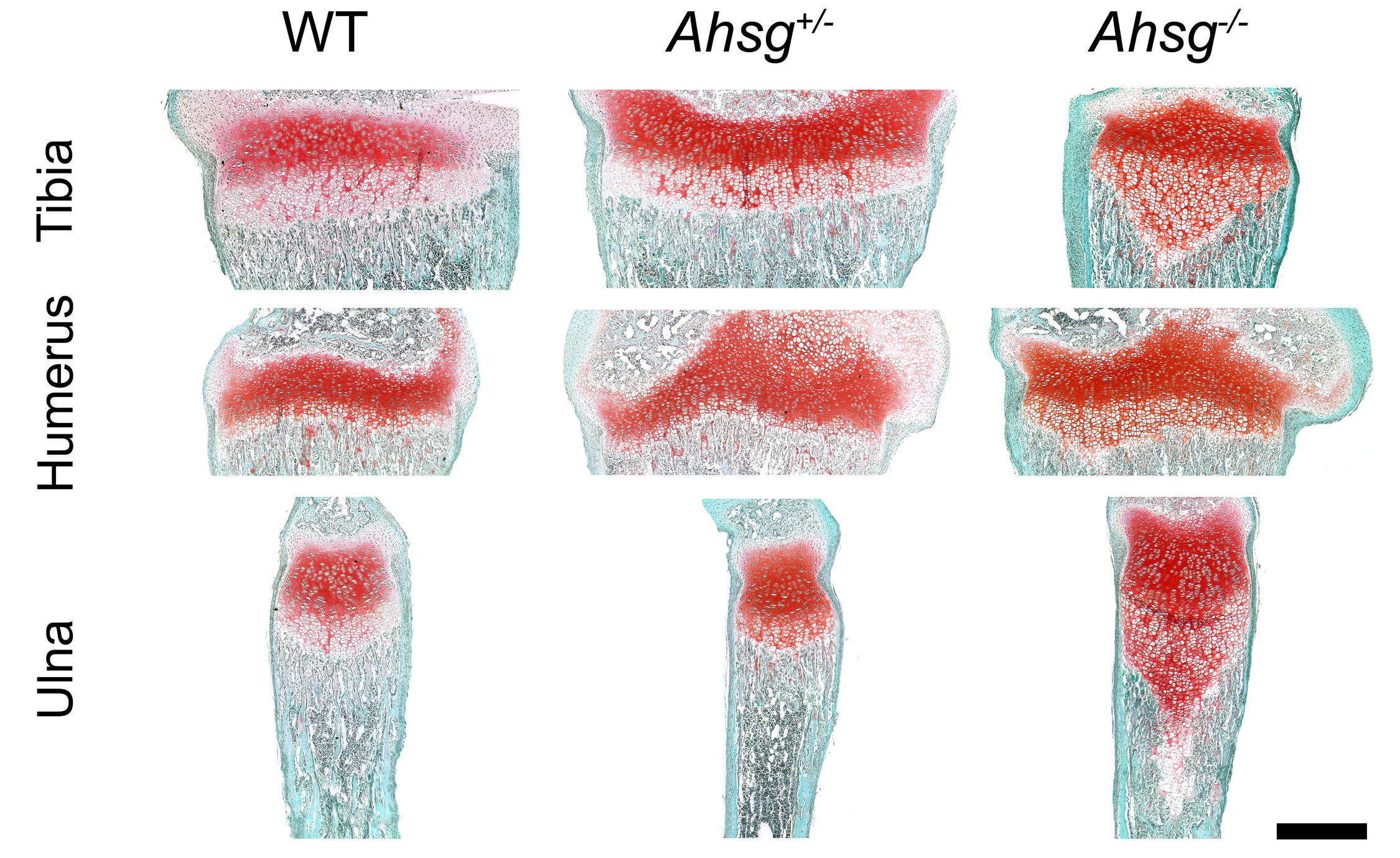

Supplement: S3 Fig — Safranin O/fast green staining of decalcified paraffin sections from 13-day-old mice show elongated and V-shaped hypertrophic zones in the growth plates of long bones. (TIF) [file pone.0187030.s003.tif]

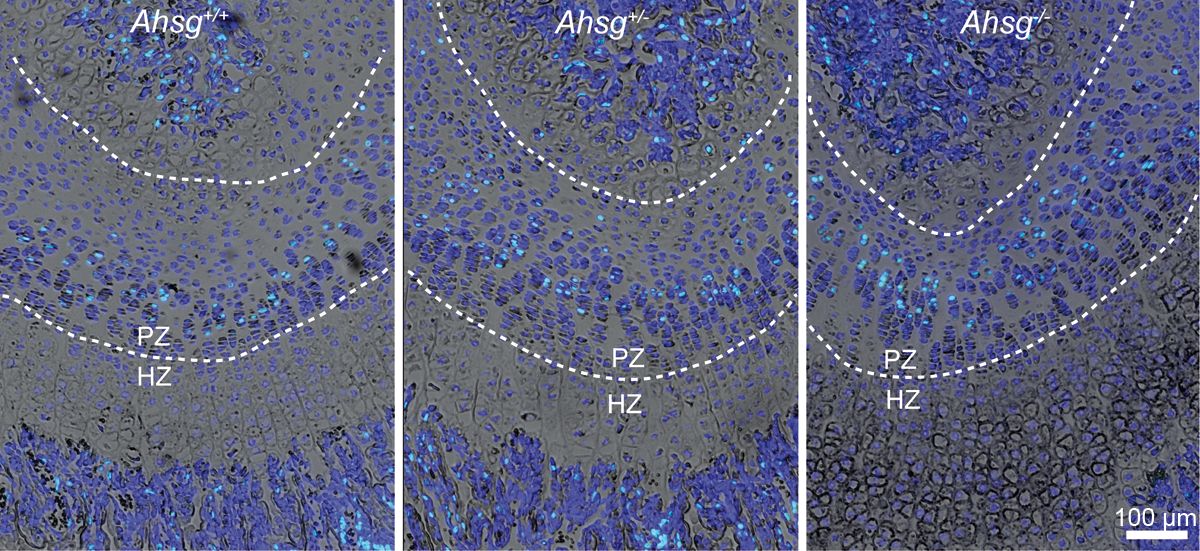

Supplement: S4 Fig — Mice were injected with EdU, sacrificed after 2 h and EdU (seen as green nuclei) was visualized on decalcified histological sections. (TIF) [file pone.0187030.s004.tif]

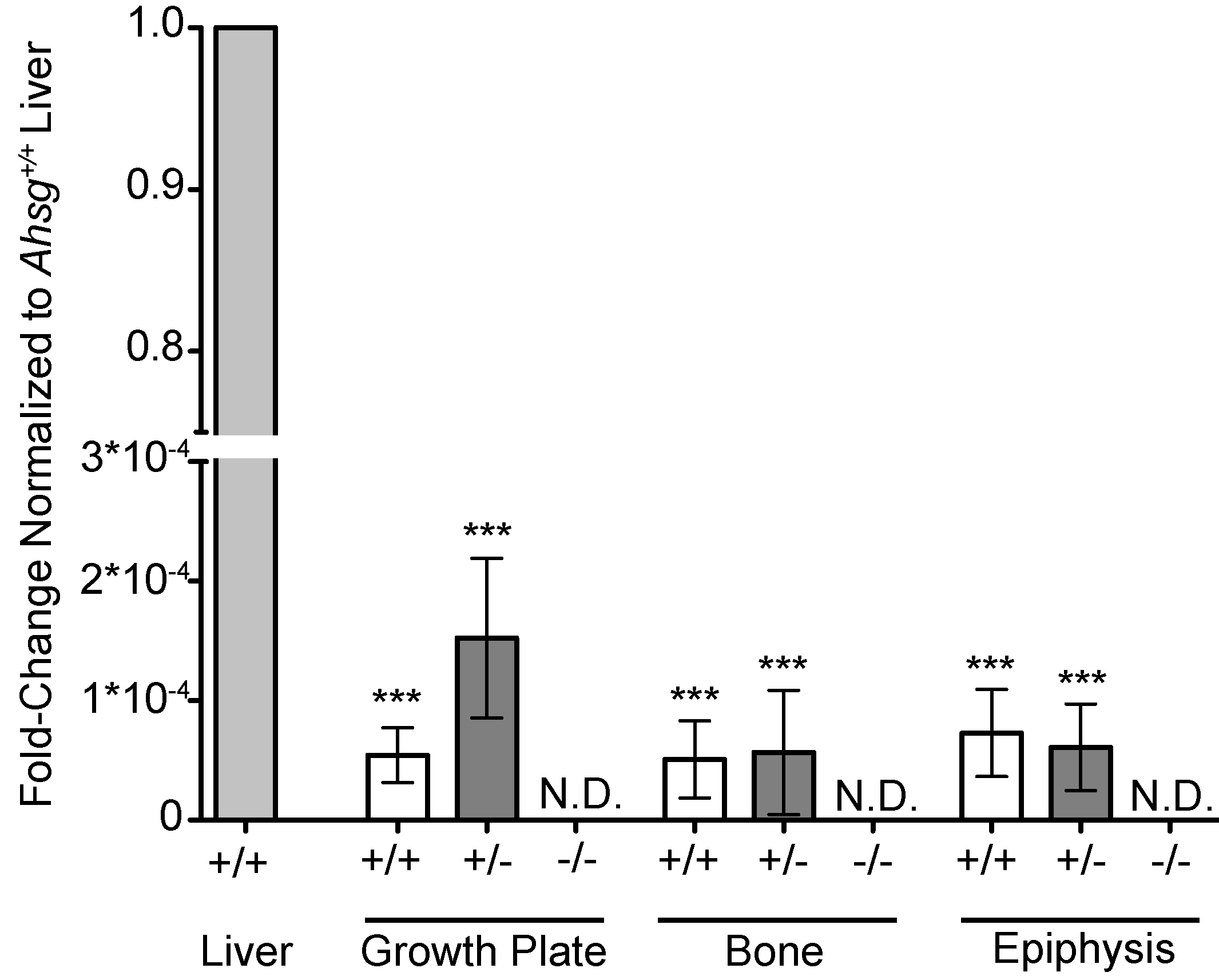

Supplement: S5 Fig — Growth plates, adjacent bone tissue and the remaining epiphyses from distal femora and proximal tibiae from 13-day-old Ahsg+/+, Ahsg+/- and Ahsg-/- mice, were manually dissected (n = 4 mice per group). Additionally, Ahsg+/+ liver samples were taken (n = 4). RNA was isolated, reverse transcribed and Ahsg mRNA expression was analyzed using qRT-PCR. Fold-changes were determined using the ΔΔCt method, using Ahsg+/+ liver as control. Expression values were compared to liver expression values using One-Way ANOVA: ***p<0.001. N.D. = not detectable. (TIF) [file pone.0187030.s005.tif]
