# Supplementary material for: Post-weaning epiphysiolysis causes distal femur dysplasia and foreshortened hindlimbs in fetuin-A-deficient mice
Source: PLoS One. 2017 Oct 31;12(10):e0187030. doi: 10.1371/journal.pone.0187030 (PMC5663435; doi:10.1371/journal.pone.0187030)
Supplement: S1 Table — Primers were designed using NCBI primer-BLAST, or taken from Primer Bank. We ensured that all primers were spanning exon-exon junctions and primed the coding region of the gene. (DOCX) [file pone.0187030.s006.docx]

| **Gene Symbol** | **Forward Primer Sequence (5´ – 3´)** | **Reverse Primer Sequence (5´ – 3´)** | **PrimerBank ID** |
| --- | --- | --- | --- |
| Gapdh^1^ | AGATGGTGATGGGCTTCCC | GGCAAATTCAACGGCACAGT | N.A. |
| Cxcl9^2^ | GGAGTTCGAGGAACCCTAGTG | GGGATTTGTAGTGGATCGTGC | 162287427c1 |
| Ifit1^2^ | GCCTATCGCCAAGATTTAGATGA | TTCTGGATTTAACCGGACAGC | 145301610c1 |
| Usp18^2^ | TTGGGCTCCTGAGGAAACC | CGATGTTGTGTAAACCAACCAGA | 6755927a1 |
| Gbp2^2^ | CTGCACTATGTGACGGAGCTA | GAGTCCACACAAAGGTTGGAAA | 6753950a1 |
| Igtp^2^ | CTCATCAGCCCGTGGTCTAAA | CACCGCCTTACCAATATCTTCAA | 31980875a1 |
| Il1b^2^ | GCAACTGTTCCTGAACTCAACT | ATCTTTTGGGGTCCGTCAACT | 6680415a1 |
| Stat1^2^ | GCTGCCTATGATGTCTCGTTT | TGCTTTTCCGTATGTTGTGCT | 328887937c2 |
| Ahsg^1^ | GCTGGCAAACTGTTCTGTGA | CCTGACTCCGTTCAACGATA | N.A. |

1: Primers designed using Primer Blast (Ye J, Coulouris G, Zaretskaya I, Cutcutache I, Rozen S, Madden TL. Primer-BLAST: a tool to design target-specific primers for polymerase chain reaction. BMC Bioinformatics. 2012;13(1):134)

2: Primer sequences taken from Primer Bank (Spandidos A, Wang X, Wang H, Seed B, Y M, D H, et al. PrimerBank: a resource of human and mouse PCR primer pairs for gene expression detection and quantification. Nucleic Acids Res. 2010;38(suppl_1):D792–9)
