# Supplementary material for: Post-weaning epiphysiolysis causes distal femur dysplasia and foreshortened hindlimbs in fetuin-A-deficient mice
Source: PLoS One. 2017 Oct 31;12(10):e0187030. doi: 10.1371/journal.pone.0187030 (PMC5663435; doi:10.1371/journal.pone.0187030)
Supplement: S2 Table — (DOCX) [file pone.0187030.s007.docx]

|  | **Number of femora measured** | | | | | |
| --- | --- | --- | --- | --- | --- | --- |
| **Age (weeks)** | **0** | **2** | **4** | **6** | **7** | **8** |
| ***Ahsg^+/+^*** | 92 | 14 | 10 | 14 | 15 | 26 |
| ***Ahsg^+/-^*** | 0 | 18 | 15 | 16 | 22 | 34 |
| ***Ahsg^-/-^*** | 68 | 27 | 10 | 16 | 14 | 33 |
