# Supplementary material for: Post-weaning epiphysiolysis causes distal femur dysplasia and foreshortened hindlimbs in fetuin-A-deficient mice
Source: PLoS One. 2017 Oct 31;12(10):e0187030. doi: 10.1371/journal.pone.0187030 (PMC5663435; doi:10.1371/journal.pone.0187030)
Supplement: S3 Table — Gene expression is given as the log2 of the mean raw spot intensity after normalizing the microarray expression data. The raw spot intensities were averaged over biological replicates. Values for Ahsg+/+ and Ahsg-/- liver expression were taken from an independent microarray analysis using the same platform. Note that the spot intensities for growth plates of all genotypes and for Ahsg-/- liver were similarly low at around 25, while the wildtype liver spot intensity was 214. (DOCX) [file pone.0187030.s008.docx]

|  | **Mean raw spot intensity** | | |
| --- | --- | --- | --- |
|  | *Ahsg^+/+^* | *Ahsg^+/-^* | *Ahsg^-/-^* |
| **Liver** | 14.029 +/- 0.075 | N.A. | 5.216 +/- 0.481 |
| **Growth plate cartilage** | 4.522 +/- 0.077 | 4.481 +/- 0.029 | 4.492 +/- 0.0252 |
