# Supplementary material for: Post-weaning epiphysiolysis causes distal femur dysplasia and foreshortened hindlimbs in fetuin-A-deficient mice
Source: PLoS One. 2017 Oct 31;12(10):e0187030. doi: 10.1371/journal.pone.0187030 (PMC5663435; doi:10.1371/journal.pone.0187030)
Supplement: S4 Table — Cibersort assumes that the gene expression data is derived from hematopoietic cells and estimates the fraction of each type of immune cell for each sample by comparing the gene expression data to established signatures of different immune cell subsets. The fractional value for 22 types of immune cells including seven T cell subtypes is listed in each row, thus for each the values add up to 1. Cibersort indicated that the fractions of T cell subtypes in the growth plates were uniformly low with no differences in growth plates derived from Ahsg+/+, Ahsg+/- and Ahsg-/- mice. Pearson correlation coefficient, root mean square deviation (MSD) and p-value confirmed the validity of the analysis. (DOCX) [file pone.0187030.s009.docx]

| **Input Sample** | **B cells naive** | **B cells memory** | **Plasma cells** | **T cells CD8** | **T cells CD4 naive** | **T cells CD4 memory resting** | **T cells CD4 memory activated** | **T cells follicular helper** | **T cells regulatory (Tregs)** | **T cells gamma delta** | **NK cells resting** | **NK cells activated** | **Monocytes** | **Macrophages M0** | **Macrophages M1** | **Macrophages M2** | **Dendritic cells resting** | **Dendritic cells activated** | **Mast cells resting** | **Mast cells activated** | **Eosinophils** | **Neutrophils** | **P-value** | **Pearson Correlation** | **RMSE** |
| --- | --- | --- | --- | --- | --- | --- | --- | --- | --- | --- | --- | --- | --- | --- | --- | --- | --- | --- | --- | --- | --- | --- | --- | --- | --- |
| **wt_Ahsg_1** | 0,086 | 0,000 | 0,067 | 0,000 | 0,042 | 0,000 | 0,000 | 0,013 | 0,000 | 0,023 | 0,020 | 0,000 | 0,021 | 0,561 | 0,003 | 0,078 | 0,000 | 0,000 | 0,086 | 0,000 | 0,000 | 0,000 | 0,000 | 0,736 | 0,808 |
| **wt_Ahsg_2** | 0,041 | 0,001 | 0,075 | 0,000 | 0,038 | 0,000 | 0,000 | 0,011 | 0,000 | 0,004 | 0,023 | 0,000 | 0,000 | 0,560 | 0,000 | 0,148 | 0,000 | 0,000 | 0,099 | 0,000 | 0,000 | 0,000 | 0,000 | 0,745 | 0,808 |
| **wt_Ahsg_3** | 0,028 | 0,009 | 0,074 | 0,000 | 0,030 | 0,000 | 0,000 | 0,018 | 0,000 | 0,009 | 0,030 | 0,000 | 0,000 | 0,533 | 0,000 | 0,182 | 0,000 | 0,000 | 0,087 | 0,000 | 0,000 | 0,000 | 0,000 | 0,742 | 0,813 |
| **het_Ahsg_1** | 0,140 | 0,011 | 0,082 | 0,000 | 0,032 | 0,024 | 0,003 | 0,000 | 0,000 | 0,014 | 0,063 | 0,000 | 0,084 | 0,388 | 0,000 | 0,067 | 0,000 | 0,000 | 0,086 | 0,004 | 0,000 | 0,002 | 0,000 | 0,663 | 0,839 |
| **het_Ahsg_2** | 0,059 | 0,004 | 0,045 | 0,000 | 0,032 | 0,000 | 0,005 | 0,000 | 0,001 | 0,033 | 0,035 | 0,000 | 0,000 | 0,665 | 0,007 | 0,057 | 0,000 | 0,000 | 0,058 | 0,000 | 0,000 | 0,000 | 0,000 | 0,764 | 0,766 |
| **het_Ahsg_3** | 0,105 | 0,000 | 0,058 | 0,000 | 0,038 | 0,002 | 0,000 | 0,000 | 0,000 | 0,029 | 0,030 | 0,000 | 0,000 | 0,521 | 0,000 | 0,121 | 0,000 | 0,000 | 0,096 | 0,000 | 0,000 | 0,000 | 0,000 | 0,737 | 0,796 |
| **ko_Ahsg_1** | 0,066 | 0,000 | 0,039 | 0,000 | 0,015 | 0,000 | 0,000 | 0,000 | 0,000 | 0,036 | 0,027 | 0,000 | 0,005 | 0,504 | 0,031 | 0,191 | 0,000 | 0,000 | 0,070 | 0,000 | 0,000 | 0,016 | 0,000 | 0,400 | 0,916 |
| **ko_Ahsg_2** | 0,024 | 0,000 | 0,025 | 0,000 | 0,019 | 0,000 | 0,006 | 0,005 | 0,000 | 0,033 | 0,036 | 0,000 | 0,000 | 0,597 | 0,026 | 0,166 | 0,000 | 0,000 | 0,060 | 0,000 | 0,000 | 0,003 | 0,000 | 0,723 | 0,778 |
| **ko_Ahsg_3** | 0,018 | 0,000 | 0,032 | 0,000 | 0,020 | 0,000 | 0,008 | 0,014 | 0,000 | 0,013 | 0,035 | 0,000 | 0,000 | 0,578 | 0,019 | 0,171 | 0,001 | 0,000 | 0,084 | 0,000 | 0,000 | 0,007 | 0,000 | 0,725 | 0,792 |
